# A randomized controlled trial with extended long-term follow-up: Quality of cervical spine motion after anterior cervical discectomy (ACD) or anterior cervical discectomy with arthroplasty (ACDA)

**DOI:** 10.1016/j.bas.2023.102726

**Published:** 2023-12-14

**Authors:** Valérie N.E. Schuermans, Anouk Y.J.M. Smeets, Inez Curfs, Henk van Santbrink, Toon F.M. Boselie

**Affiliations:** a Dept. of Neurosurgery, Maastricht University Medical Center, Maastricht, the Netherlands; b Dept. of Neurosurgery, Zuyderland Medical Center, Heerlen, the Netherlands; c CAPHRI School for Public Health and Primary Care, Maastricht University, Maastricht, the Netherlands; d Dept. of Orthopaedic Surgery and Traumatology, Zuyderland Medical Center, Heerlen, the Netherlands

**Keywords:** Cervical spine, Motion analysis, Range of motion, Arthroplasty, Fusion

## Abstract

**Introduction:**

In previous research, a consistent sequence of segmental contributions during dynamic extension X-rays of the cervical spine was observed in 80–90% of healthy participants.

**Research question:**

To investigate whether this previously defined ‘normal’ sequence of segmental contributions was present in patients who underwent anterior cervical discectomy with arthroplasty (ACDA) or anterior cervical discectomy (ACD).

**Materials & methods:**

A randomized controlled trial with extended follow-up was conducted. Patients with single level cervical degenerative radiculopathy with a surgical indication were included and randomized. Dynamic X-ray recordings were made before surgery, one-year post-operative, and at long term follow-up.

**Results:**

A total of 27 patients were included, three in an ACDA pilot group and 24 were randomized to receive ACDA (N = 12) or ACD (N = 12). A total of 20 patients were available for follow-up. Preoperatively, 16.7% of patients in the ACDA group and 58.3% of patients in the ACD group showed a normal sequence. One-year post-operative, 66.7% showed a normal sequence in the ACDA group versus 30.0% in the ACD group (p = 0.036). After an average of 11-years follow-up, a normal sequence was observed in 9.1% of patients in the ACDA group and in none of the patients in the ACD group (p = 0.588).

**Discussion & conclusion:**

These findings suggest that while ACDA can restore and preserve a normal sequence of segmental contributions in the short term, this effect is not maintained in the long term. Throughout the process of ageing, not only the quantity, but also the quality of motion changes.

## Highlights

• Preoperatively, only 37.5% with cervical degenerative disc disease exhibit the typical movement pattern, contrasting with 80–90% in healthy, young individuals.
• ACDA restores motion pattern in 80% post-op at one year, surpassing ACD's 30%.
• Long-term follow-up (9–15 years) reveals that neither ACDA nor ACD sustains 'normal' motion patterns, regardless of fusion presence.
• Single-level radiculopathy patients have favorable clinical outcomes, irrespective of surgical technique.
• ACDA raises TROM, sROM at index and upper levels significantly compared to ACD, short- and long-term.

## Abbreviations

ACD =: Anterior cervical discectomy
ACDA =: Anterior cervical discectomy with arthroplasty
ACDF =: Anterior cervical discectomy and fusion
ASP =: Adjacent Segment Pathology
KS =: Kellgren Score
METC =: Medical Ethics Review Committee
NDI =: Neck Disability Index
RCT =: Randomized controlled trial
ROM =: Range of motion
sROM =: Segmental range of motion
tROM =: Total range of motion
SF-36 =: Short Form 36
VAS =: Visual Analogue Scale

## 1 Introduction

The underlying mechanism of adjacent segment pathology (ASP) after cervical fusion remains a matter of debate. In addition to natural progression of degeneration, compensation for the loss of motion in the fused segment is thought to cause overstraining of the adjacent segments (Helgeson et al., 2013; Seo and Choi, 2008; Eck et al., 2002). The extent to which cervical spine kinematics, surgery-induced fusion, and natural progression of degeneration play a role in the development of ASP remains unclear (Boselie et al., 2013, 2015).

Anterior cervical discectomy with arthroplasty (ACDA) was developed in an effort to reduce the incidence of clinical ASP by preserving motion of the operated segment. Previously conducted research in patients with radiculopathy and/or myelopathy has shown that short term clinical and radiological outcomes are similar between ACDA and anterior cervical discectomy with fusion (ACDF) or without fusion (ACD) (Helgeson et al., 2013; Vleggeert-Lankamp et al., 2019; Zhang et al., 2015; Findlay et al., 2018). However, additional adjacent segment surgery rates seemed to be lower for ACDA, for both single- and multi-level surgeries (R and. C, 2016; Kong et al., 2016). The difference in additional adjacent segment surgery rates between ACDA and ACDF increases with a longer-follow-up time (Schuermans et al., 2022; Schuermans et al.a). Because of the inconsistency in reported risk factors of ASP, the role of preserved kinematics by ACDA in preventing ASP remains unclear.

Multiple studies have assessed differences in range of motion (ROM) after motion preserving surgery and fusion surgery (Findlay et al., 2018; Byvaltsev et al., 2020). They generally show preserved motion after ACDA, defined by a similar segmental ROM (sROM) compared to the pre-operative sROM, in contrast to a decrease in postoperative sROM after fusion. sROM is measured by the amount of sagittal rotation in a segment between the maximum flexion and the maximum extension position of the entire cervical spine. There are several methods of measuring and calculating the sROM, but they are all limited by high intra- and interindividual variability (Lind et al., 1989; Dvorak et al., 1991; Bhalla and Simmons, 1969; AHO et al., 1955; Van Mameren et al., 1990). Individuals show high variations in sROM when measured at different timepoints, which makes sROM an unreliable outcome measure for use in individual subjects (Van Mameren et al., 1990). This is also demonstrated by Anderst et al., who have observed altered motion paths in the adjacent segments after anterior arthrodesis, which could not have been identified by looking at end-point ROM alone (Anderst et al., 2014). Moreover, the maximum amount of rotation of a segment can be present within the motion path, and would be missed if it is only measured by comparing the maximum flexion and extension positions (Boselie et al., 2017a).

In a previous study by our group, the sequence of segmental contributions during flexion and/or extension of the entire cervical spine in dynamic X-ray recordings was found to be a consistent parameter for cervical spine motion (Bogduk and Mercer, 2000; van Mameren, 1988). A consistent motion pattern was observed in 80–90% of 20 healthy participants during the second phase of extension, in which the sequence of segmental contributions in the lower cervical spine was from cranial to caudal (Boselie et al., 2017a, 2017b; Reinartz et al., 2009). The first peak of segmental rotation was in C4–C5, followed by C5–C6 and finally in C6–C7. Presence of this motion pattern was shown to have high sensitivity and specificity (90% and 85%, respectively) in differentiating between healthy subjects and pre-operative patients with symptomatic CDDD (Boselie et al., 2017a).

Therefore, the primary aim of the current randomized controlled trial (RCT) is to analyze the pre- and postoperative sequence of segmental contributions of the lower cervical spine; before ACD or ACDA surgery, one year after surgery and at long-term. The secondary objectives of this study involve evaluating clinical parameters such as pain and subsequent surgical interventions, alongside radiological criteria including fusion, progression of degenerative changes, and range of motion.

## 2 Methods

### 2.1 Study design

This is an RCT with extended long-term follow-up. Patients referred to our neurosurgical department with radiculopathy due to single level CDDD with a soft disc herniation and eligible for surgery were asked to participate. Patients were randomized for ACD (N = 12) or ACDA (N = 12). Randomization was performed in a 1:1 allocation rate with no fixed block size using unmarked, non-sequential, sealed, opaque envelopes. Patients and caregivers were not blinded to allocation. Assessment of the radiological data was not blinded, since absence or presence of a prosthesis is clearly visible. At the start of the trial, ACD was chosen as the standard of care in our center. A pilot group of 3 patients undergoing ACDA was included to correct for a learning curve.

Patients underwent surgery between December 2008 and September 2014. All patients were operated in the same center by a single experienced spine surgeon. Flexion and extension cinematographic recordings of the cervical spine were made before surgery and at one-year post-operatively. Subsequently, all included patients were contacted for the extended long-term follow-up. The RCT has been approved by the local institutional medical ethical committee (Medical Research Ethics Committee Maastricht UMC+, METC 06-1-098) and has been registered on clinicaltrials.gov (NCT00868335). The long-term follow-up of the study was approved by the medical ethical committee of the Zuyderland Medical Center (Z2020101) and registered before the start of the study (NCT04545983). Informed consent was acquired from all participants for both the RCT and the long-term follow-up.

### 2.2 In- and exclusion criteria

Patients with an indication for ACD because of a monoradicular syndrome at C5–C6 or C6–C7 with corresponding pathology on MRI were included. Strict inclusion criteria were enforced because at the start of the study it was believed that disc arthroplasty was only suitable for this specific patient population.

Before the randomization 3 patients were operated in a pilot group (ACDA), resulting in inclusion of a total of 27 patients. These patients were also asked to participate in the extended follow-up.

Inclusion criteria.

• An indication for ACD because of a monoradicular syndrome due to CDDD at C5–C6 or C6–C7, with corresponding pathology on MRI.
• Aged 18–55 years.
• Able to actively perform flexion/extension movements.
• Able to read the information form and sign informed consent.

Patients were excluded based on the following criteria.

• Previous surgery to the cervical spine.
• Ongoing or active infection.
• Previous or actual tumorous processes in the cervical region.
• Pregnancy.
• Previous radiation therapy in the cervical region.

### 2.3 Procedure

In both techniques the disc space contents were resected through a right-sided anterior surgical approach. The endplates were prepared with curettes and the disc space content was removed. The posterior longitudinal ligament was opened and the dura was visualized to ensure adequate decompression. In the ACD group only a simple discectomy without implantation of a cage was performed and in the ACDA group a disc prosthesis was implanted after the discectomy. The prosthesis used in this RCT is the Activ-C (B. Braun Aesculap, Germany). This was done in accordance with the manufacturer's protocol for implantation and endplate preparation. In both groups, the wound was closed in layers, after a prevertebral wound drain was placed. Post-surgical care was the same for both groups. Patients received 24 h of antibiotic prophylaxis and the prevertebral wound drain was removed the day after surgery. Patients could use analgesics postoperatively and they were allowed to mobilize immediately. The day after surgery a physical therapist provided patients with standardized exercises. Patients were not routinely referred to a physical therapist and no cervical collar was used.

### 2.4 Sample size calculation

The power-calculation was made based on expected rate of fusion, as it was hypothesized that fusion and abnormal motion correlate one to one. Therefore, we hypothesized that ACDA would maintain normal sequence of motion, whereas ACD with fusion does not. Based on available literature at that time, a fusion rate of 15% was assumed for ACDA (reported range of 0–20%) versus a fusion rate of 70–80% for ACD. At an α of 0.05 and power (β) of 80% the number of patients for the previous study to detect a 60% difference in the primary outcome, the presence of a normal sequence of segmental contributions, was 10 per group. The assumptions made above were in line with the percentages that were actually found one year after surgery in this RCT and in line with sample sizes that were previously used in the analysis of this type of cinematographic recording (Boselie et al., 2015, 2017a).

### 2.5 Data acquisition

Cinematographic recordings were made following the same protocol, both for the RCT and the long-term follow-up. Detailed information concerning the acquisition of radiographs can be found in the published study protocol (Eck et al., 2002).

Clinical and radiological data were collected preoperatively, at one year postoperative, and at long-term follow-up [range 9–15 years]. Both tROM and sROM were calculated at all timepoints to increase comparability with existing literature. Clinical data included the neurological status, visual analogue score (VAS) scores for arm and neck pain, Neck Disability Index (NDI), quality of life according to the Short Form 36 (SF-36), and outcomes according to Odom's outcome criteria. Patients were also asked whether they developed new symptoms of cervical radiculopathy and/or myelopathy and if they underwent additional surgeries to the cervical spine.

### 2.6 Data analysis

Images were analyzed using computer software that uses an image recognition-based algorithm to follow motion of the vertebrae during complete flexion and extension developed in Wolfram Mathematica (Wolfram Research, Inc., Version 9.0, Champaign, IL). This method has been validated previously, with a good intra- and interobserver reliability (Van Mameren et al., 1990; Boselie et al., 2017a; Schuermans et al.b; Schuermans et al., 2023). According to the previously described method, the sequence of motion was determined on graphs representing the segmental rotation against the cumulative rotation of the segments C4 to C7 (Eck et al., 2002). Fusion was defined as bony bridging between two or more adjacent vertebral segments as assessed on radiographic imaging, or less than 2° sROM (Boselie et al., 2015; NCT04545983, 2020).

Continuous data were analyzed using an independent samples *t*-test unless normality was not met, in which case a Mann-Whitney *U* test was used. Dichotomous data were analyzed using a Chi-squared analysis. If the criteria for a Chi-squared analysis were not met (i.e., less than five expected samples per field), the Fisher's exact test was used. For all tests, statistical significance was defined as p < 0.05. Statistical analyses were carried out using IBM SPSS statistics 27 (Cooperation).

## 3 Results

### 3.1 Population

A total of 27 patients were included, of which 3 were operated in a pilot group and 24 were operated in the RCT. The pilot group was included in the motion analysis as it was a pilot for surgical learning curve, which is not thought to influence kinematics. A sensitivity analysis was subsequently conducted, omitting the pilot group from the dataset, and the outcomes remained consistent. Table 2 displays the exact number of patients analyzed per group at each timepoint.

**Table 1:** Baseline characteristics of study population, including the pilot group. M = male, F = female, SD = standard deviation.

Index surgery	Number of patients	Level of index surgery (N = )		Sex (M/F)	Age at baseline (years ± SD)	Long term follow-up (years ± SD)
**ACDA**	15	C5–C6	11 (73.3%)	9/6	41 ± 8.1	11.7 ± 2.2
		C6–C7	4 (26.7%)			
**ACD**	12	C5–C6	6 (50%)	7/5	42 ± 5.7	11.0 ± 0.9
		C6–C7	6 (50%)

**Table 2:** Radiological outcomes at 1 year and extended follow-up as measured in the study population, including the pilot group (Patient 1,2 and 3). Presence of the consistent, previously defined ‘normal’, sequence C4–C5, followed by C5–C6 and then C6–C7 during the second half of extension in dynamic X-ray recordings. ACD = anterior cervical discectomy, ACDA = anterior cervical discectomy with arthroplasty.

Patient	Index	Level of index surgery	Fusion at index level		Additional surgery	‘Normal’ sequence (±)		
			1 year	Long-term (9–15 years)		Pre-operative	1 year	Long term (9–15 years)
1	ACDA	C5–C6	–	–	–	–	–	n.a.
2	ACDA	C5–C6	–	–	–	+	–	–
3	ACDA	C6–C7	–	–	–	–	+	–
4	ACDA	C5–C6	–	+	–	–	–	–
5	ACDA	C5–C6	n.a.	–	–	–	n.a.	–
6	ACD	C5–C6	–	n.a.	n.a.	+	–	n.a.
7	ACD	C6–C7	–	n.a.	n.a.	+	+	n.a.
8	ACD	C5–C6	–	–	–	+	+	–
9	ACD	C6–C7	–	+	–	+	+	n.a.
10	ACD	C6–C7	+	+	ACDF C6–C7 ACDF + plate construct C5–C6	+	–	–
11	ACD	C6–C7	+	n.a.	n.a.	+	–	–
12	ACDA	C6–C7	–	–	–	+	+	–
13	ACDA	C5–C6	–	–	–	–	+	–
14	ACD	C6–C7	–	–	–	–	–	–
15	ACDA	C5–C6	–	+	–	–	+	–
16	ACD	C5–C6	n.a.	+	ACDF C4–C5	–	n.a.	–
17	ACDA	C5–C6	–	–	–	–	+	–
18	ACD	C6–C7	+	+	–	–	–	–
19	ACD	C5–C6	+	+	–	–	–	–
20	ACD	C5–C6	+	n.a.	–	+	–	n.a.
21	ACDA	C5–C6	n.a.	n.a.	n.a.	–	n.a.	n.a.
22	ACDA	C5–C6	–	–	–	–	+	+
23	ACDA	C5–C6	–	+	–	–	+	–
24	ACDA	C5–C6	–	–	Dorsal foraminotomy C6–C7	–	–	–
25	ACD	C5–C6	+	n.a.	n.a.	–	n.a.	n.a.
26	ACDA	C6–C7	n.a.	n.a.	n.a.	–	n.a.	n.a.
27	ACDA	C6–C7	–	n.a.	n.a.	–	+	n.a.
**ACD**		**10 C5–C6** **2 C6–C7**	**6/10 (60%)**	**4/6 (71.4%)**	**2/8 (25.0%)**	**7/12 (58.3%)**	**3/10 (30%)**	**0% (0/7)**
**ACDA**		**4 C5–C6** **4 C6–C7**	**0/13 (0%)**	**4/13 (30.7%)**	**1/12 (8.3%)**	**2/15 (13.3%)**	**8/12 (66.7%)**	**9.1% (1/11)**
**p-value**			0.002*	**0.053**	**0.270**	**0.398**	0.036*	**0.588**

At baseline, the average age of the patients was 41.4 years [range 31–51] and 11/27 (41%) of patients were female. There were no statistically significant differences in baseline characteristics between the ACDA (N = 15) and ACD (N = 12) groups. Baseline characteristics of the included participants are outlined in Table 1. All patients received the allocated treatment.

In total, 24/27 of randomized patients were available for follow up one year after surgery. A total of 20 patients, including the pilot group, was available for long-term follow-up at an average of 11-years post-operative [range 9–15 years]. Of the patients available for long term follow-up, 12 patients previously underwent ACDA, and 8 underwent ACD. Of these 20 patients, two patients were not available for radiological follow-up, but were able to fill out the clinical outcome questionnaires. A recent X-ray was available for one of these patients which was used to assess fusion status and degeneration even though the dynamic recordings was missing.

At an average of 11 years follow-up, fusion was present in 4/13 (30.7%) of the ACDA patients, and in 4/6 (71.4%) of the ACD patients. In the ACDA group, 8.3% of patients underwent additional surgeries at adjacent segments, whereas 25% of ACD patients underwent additional surgery at an adjacent segment (p = 0.027). No complications occurred in either of the groups.

### 3.2 Primary outcome

#### 3.2.1 Sequence of segmental contributions

Preoperatively 2/12 (16.6%) of patients in the ACDA group and 7/12 (58.3%) of patients in the ACD group had a normal sequence of segmental contributions (p = 0.398). At one year after surgery 8/12 (66.7%) showed a normal sequence of segmental contributions in the ACDA group versus 3/10 (30.0%) in the ACD group (p = 0.036) [Table 2]. At long-term follow-up, a normal sequence was observed in (1/11) 9.1% patients in the ACDA group and in 0/7 patients in the ACD group.

**Fig. 1:**
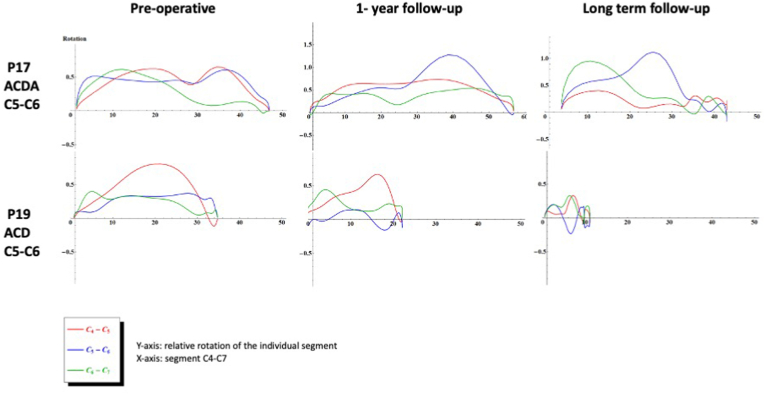
Representative graphs of two individual patients undergoing ACDA and ACD. The x-axis represents the extension motion over time. The y-axis represents relative rotations. The sequence of segmental contributions is shown at three timepoints; pre-operative, after 1-year follow-up and after long-term follow-up. The red line represents the relative rotations of the segments C4-C5, the blue line represents C5-C6 and the green line represents C6-C7. ACD = Anterior cervical discectomy. ACDA = Anterior cervical discectomy and arthroplasty.

The two patients in the ACDA group with a normal sequence of segmental contributions preoperatively, still had a normal sequence at one year [Table 2]. The normal sequence was not preserved in these patients at long-term follow-up. Representative graphs for the ACDA group and the ACD group are shown in Fig. 1, the graphs from all patients are available in appendix 1.

### 3.3 Secondary outcomes

#### 3.3.1 Range of motion

At baseline, the mean total ROM (tROM) from C4–C7 was similar in both groups, with an average of 44.1° and 43.4° for ACD and ACDA respectively [Table 3]. At baseline, no statistically significant differences were seen in sROM of the index level, or at the adjacent levels.

**Table 3:** Range of motion in each analyzed segment and total range of motion of block C4–C7 at 1 year and at long-term follow-up. Statistically significant differences are indicated with an asterisk (*). tROM = total range of motion, sROM = segmental range of motion, AS = adjacent segment, SD= Standard Deviation, ACD = anterior cervical discectomy, ACDA = anterior cervical discectomy with arthroplasty.

		tROM (°) C4–C7	p-value	sROM index level	p-value	sROM upper AS	p-value	sROM lower AS	p-value
**Preoperative**	**ACD** ± **SD (N** = **12)**	44.1 ± 10.2	0.841	15.1 ± 4.5	0.587	15.1 ± 4.5	0.324	14.5 ± 3.9	0.356
	**ACDA** ± **SD (N** = **15)**	43.4 ± 8.9		14.2 ± 3.7		15.1 ± 3.3		12.5 ± 4.3	
**1-year**	**ACD** ± **SD (N** = **10)**	35.1 ± 6.6	0.008 *	4.5 ± 3.3	<0.001 *	14.0 ± 3.0	0.024 *	17.8 ± 5.3	0.164
	**ACDA** ± **SD (N** = **13)**	45.1 ± 9.1		13.8 ± 5.0		16.8 ± 2.6		13.8 ± 4.1	
**Long-term [9**–**15 years]**	**ACD** ± **SD (N** = **7)**	22.2 ± 7.0	0.018 *	2.1 ± 2.2	0.005 *	7.4 ± 3.0	0.005 *	13.0 ± 6.6	0.126
	**ACDA** ± **SD (N** = **11)**	31.8 ± 7.7		9.4 ± 5.7		13.3 ± 4.4		7.1 ± 4.8	

At one-year, mean sROM at the index level was statistically significant higher in the ACDA group compared to the ACD group, 13.8° versus 4.5° respectively. This is also observed at long-term follow-up, with an average of 9.4° at the index level of the ACDA group versus 2.1° at the index level of the ACD group.

The sROM of the lower adjacent segments did not differ between the two groups at 1-year, nor at long-term follow-up. The sROM of the upper adjacent segment was significantly higher in the ACDA group than in the ACD group, after 1 year and at long-term follow-up. SROM in each individual segment and the total ROM from C4–C7 were significantly lower at long-term compared to 1-year post-operative [appendix 2].

#### 3.3.2 Degeneration

The level of degeneration was assessed in all 27 patients at baseline and in 19 patients at long-term follow-up [Table 4]. Kellgren's score of the index level was not included in the analysis as it is either fused or has an implant in situ. The level of degeneration was significantly higher in segment C2–C3 and C3–C4 after ACD compared to ACDA, but not at the other segments. Degeneration in the adjacent segments was comparable between the two groups at long-term follow-up. However, when comparing the change in degeneration from pre-operative to long-term follow-up, the lower adjacent segment showed significantly more degeneration after ACDA when compared to ACD.

**Table 4:** Level of degeneration per segment at long-term follow-up, assessed according to Kellgren's Scale. Statistically significant differences are indicated bold with an asterisk (*). KS=Kellgren's Scale. SD= Standard Deviation, ACD = anterior cervical discectomy.

Kellgren Scores (KS) of the individual segments	Pre-operative			Long-term follow-up		
	ACD KS ± SD (N = 12)	ACDA KS± SD (N = 15)	p-value	ACD KS ± SD (N = 7)	ACDA KS ± SD (N = 12)	p-value
**C2–C3**	1.2 ± 0.4	1.1 ± 0.3	0.628	1.4 ± 0.5	1.0 ± 0.0	0.014*
**C3–C4**	1.0 ± 0.0	1.2 ± 0.4	0.231	1.0 ± 0.5	1.3 ± 0.0	0.049*
**C4–C5**	1.1 ± 0.3	1.2 ± 0.4	0.605	1.7 ± 1.1	1.2 ± 0.4	0.239
**C5–C6**	1.7 ± 0.4	1.5 ± 0.6	0.054	2.6 ± 0.9	2.0 ± 0.9	0.165
**C6–C7**	1.3 ± 0.6	1.3 ± 0.6	1.000	2.0 ± 1.4	1.6 ± 0.9	0.594
**Upper adjacent segment**	1.3 ± 0.5	1.3 ± 0.4	1.000	2.2 ± 1.2	1.3 ± 0.5	0.154
**Lower adjacent segment**	1.2 ± 0.4	1.2 ± 0.4	1.000	1.3 ± 0.6	1.7 ± 0.7	1.000
**index segment**	1.8 ± 0.4	1.6 ± 0.6	0.147			

#### 3.3.3 Clinical outcomes

There were no significant differences in VAS, NDI or SF-36 at baseline between the ACD and ACDA group. At one year follow up there were no significant differences for any of the clinical outcomes [Table 5]. Patients in both groups had showed significant improvement in clinical outcomes compared to baseline.

**Table 5:** Clinical outcomes at last moment of follow-up. Statistically significant differences are indicated with an asterisk (*). VAS=Visual analogue scale, NDI = Neck Disability Index, SD= Standard Deviation, ACD = anterior cervical discectomy, ACDA = anterior cervical discectomy with arthroplasty.

		NDI (%) (±SD)	VAS neck (±SD)	VAS arm (±SD)	SF-36 Mental component (±SD)	SF-36 Physical component (±SD)	Odom's criteria
**Pre-operative**	**ACD (N** = **12)**	57.6 (±19.2)	5.5 (±2.7)	6.4 (±2.3)	38.0 (±13.9)	37.6 (±8.1)	n.a.
	**ACDA (N** = **12)**	57.1 (±18.9)	5.8 (±2.9)	6.7 (±1.9)	35.6 (±12.0)	38.3 (±5.4)	n.a.
	**P-value**	0.954	0.777	0.344	0.658	0.804	
**1 year**	**ACD (N** = **10)**	31.1 (±19.4)	2.5 (±3.8)	1.9 (±2.8)	44.3 (±13.7)	49.2 (±9.1)	Excellent = 40%
							Good = 30%
							Fair = 20%
							Poor = 10%
	**ACDA (N** = **10)**	33.8 (±14.9)	3.1 (±3.7)	0.6 (±0.8)	44.8 (±11.8)	44.3 (±10.9)	Excellent = 30%
							Good = 70%
							Fair = 0%
							Poor = 0%
	**P-value**	0.734	0.419	0.195	0.309	0.990	0.586
**9**–**15 years**	**ACD (N** = **8)**	15.0 (±8.4)	3.5 (±2.9)	3.7 (±2.4)	67.5 (±27.0)	61.4 (±24.8)	Excellent = 12.5%
							Good = 75%
							Fair = 12.5%
							Poor = 0
	**ACDA (N** = **12)**	9.6 (±9.6)	2.7 (±3.5)	2.0 (±2.3)	66.0 (±26.5)	62.8 (±26.5)	Excellent = 41.6%
							Good = 41.6%
							Fair = 16.7%
							Poor = 0
	**P-value**	0.202	0.610	0.148	*0.762*	*0.759*	0.194

At long-term follow-up, clinical outcomes were assessed in 20 patients and also no statistically significant differences were found.

## 4 Discussion

In this RCT, we investigated the sequence of segmental contributions in patients randomly allocated to ACDA or ACD pre-operatively, one-year post-operative and at long-term follow up. In 8/12 (66.7%) of patients with ACDA a consistent motion pattern was observed one-year after surgery, which was only present in 3/10 (30%) of the ACD patients. However, at long-term follow up neither the fused nor the mobile cervical spines moved according to this consistent pattern.

We recently found that this consistent sequence, which was present in asymptomatic young individuals (average age 23), disappears over the years in elderly patients (55–70 years) (Schuermans et al., 2023). This altered contribution of the cervical segments during extension in elderly did not appear to be caused by degeneration or clinical condition. Therefore, the change in motion pattern seems to be associated with ‘normal’ aging, which means that not only the quantity, but also the quality of motion decreases over the years. The influence of fusion surgery or cervical arthroplasty might thus affect elderly differently.

Yeni et al. also describe a difference in adjacent segment motion patterns when comparing ACDF and ACDA at 2-years follow-up, they observed that this pattern disappears after 6.5 years follow-up (Yeni et al., 2022). Their findings at 2-year follow-up were similar to those of Anderst et al. and Wang et al. (Anderst et al., 2014; Wan et al., 2021). Even though Yeni et al. have used a different technique, a biplanar x-ray with 3D model tracking, and assessed foraminal rotations and translations in patients undergoing fusion and ACDA, our findings appear to be similar. In the short term a consistent motion pattern seems to be present, although at long term follow-up, this disappears. Given our recent observations in asymptomatic elderly individuals this appears to be a more general evolution, not something that specifically affects surgical patients. To our best knowledge, no other studies comparing motion patterns after anterior cervical spine surgery are available (Anderst et al., 2014; Wan et al., 2021).

In this study, an obvious and statistically significant decrease in ROM is observed in both ACD and ACDA patients after 9–15 years. ACDA significantly preserves sROM over the years in comparison to ACD, both in the index and upper adjacent levels. However, a similar decrease in sROM of the lower adjacent level is seen in patients with ACD and ACDA.

It has previously been described that fusion of a segment may increase the sROM at both the superior and inferior adjacent level, as a compensation for loss of motion (Seo and Choi, 2008; Eck et al., 2002). No such increase was found in the adjacent levels of both the ACD and ACDA groups at long-term follow-up.

A recent systematic review and meta-analysis shows that sROM initially improves after ACDA, but at the long-term (average of 5-years), sROM diminishes back to values similar to those preoperatively, which is in line with our findings (Zavras et al., 2022). A recent publication by Wu et al. also demonstrated that ROM increases after ACDA in younger patients, but decreases in elderly patients after 2 years (Wu et al., 2019). Possibly, this is related to the restored motion patterns on the short term, which diminishes at long-term follow-up. The influence of ageing might be underestimated in previous studies investigating cervical spine motion after surgical treatments.

A difference is also found in the degree of degeneration. Degeneration at baseline was similar between the two groups. A higher degree of radiological degeneration in the lower adjacent level was seen in the ACDA group. This might be biased by the fact that only 3 patients with ACD at index level C5–C6 were available, meaning that the KS in the lower adjacent segment was only scored in 3 patients, as C7–Th1 was not visible in those with index level C6–C7.

The use of different types of implants might also contribute to the heterogeneity in findings described in literature. As Colman et al. observed, a significant difference in ROM can be attributed to the design of the prothesis. Our study only assesses one type of prosthesis and can therefore not provide insights in the comparison of different prosthesis designs.

Pain has been described to be a cause of altered motion patters in the cervical spine (Tsang et al., 2014). Altered motion could be a way of the body to avoid pain due to radicular compression or to avoid motion in a degenerated disc. Absence of a normal sequence of segmental contributions in these patients preoperatively might be caused by neck pain, as indicated by high VAS- and NDI-scores. When comparing the patients with and without a normal sequence of segmental contributions preoperatively, there were no significant differences in VAS score for arm pain (p = 0.579), or VAS score for neck pain (p = 0.909). Simply the presence of symptoms, not the severity, might be the determining factor.

Some limitations need to be considered when interpreting the results of this study. First, this study is powered on the radiological primary outcome: presence of normal motion patterns 1-year post-operative. All other findings should be interpreted with caution as the sample size is small for an RCT. Patient reported outcomes were not the primary focus of this study but were evaluated to possibly detect larger discrepancies with other studies that did focus on this outcome. Although the sample size was small, resulting secondary outcome parameters were comparable with trials powered for these outcome parameters. This observation suggests that these clinical parameters are representative.

A discrepancy was observed in the baseline motion sequences between the two groups. Unfortunately, this is the disadvantage of 1:1 randomization in smaller groups when block randomization is not suitable. At baseline, the ACD group exhibited a higher percentage of normal motion compared to the ACDA group. However, postoperatively, this ratio was reversed, with the ACDA group displaying an improvement in motion patterns across most parameters, while consistent motion patterns disappeared in the ACD group. This reversal of effects constitutes the primary outcome of this study, and we maintain that the results are valid despite the discrepancies in baseline motion patterns.

The group sizes differ at the timepoints as initially the pilot group was not Included in the RCT, but was included for the extended follow-up. This is why we chose to also include and analyze the pilot group at baseline and 1-year post-operative for an adequate comparison. A sensitivity analysis was conducted, excluding the pilot group, and it reaffirmed the validity of the results, whether with or without the inclusion of the pilot group.

Only rotation in the sagittal plane is investigated. Analysis of translation in the sagittal or coronal plane, lateral flexion, and axial rotation are not evaluated. However, the method of motion pattern analysis used in this study gives more information than the traditionally made function radiographs. This allows for a more thorough analysis of mobility of the target level, as well as analysis of motion patterns of the cervical spine. Although ACD is not commonly performed nowadays, this study gives new insight concerning cervical spine kinematics after fusion surgery compared to motion preserving surgery. The primary issue concerning ACD is the post-operative sagittal alignment. This was not investigated in this study, but higher rates of clinical ASP have been observed after ACD, concomitant with an increased segmental kyphosis at the index level, which should be taken into account when interpreting the results of this study (Xie and Hurlbert, 2007; Martins, 1976; Hauerberg et al., 2008).

## 5 Conclusion

In this study, we observed that ACDA initially restores and preserves a sequence of segmental contributions similar to that of young, asymptomatic individuals. However, at the long-term not only the quantity, but also de quality of motion changes, irrespective of undergoing ACDA or ACD at the index level. In this relatively small RCT, a higher rate of adjacent level surgery after ACD was found, but this was not statistically significant. Possibly, the fact *that* ACDA moves transcends the importance of *how* it moves in the prevention of ASP. In this perspective, the potential clinical relevance of a normal sequence of segmental contributions in itself is doubtful.

## Ethics approval and consent to participate

The RCT has been approved by the local institutional medical ethical committee (Medical Research Ethics Committee Maastricht UMC+, METC 06-1-098) and has been registered on clinicaltrials.gov (NCT00868335). The current study was approved by the medical ethical committee of the Zuyderland Medical Center (Z2020101) and registered before the start of the study (NCT04545983). The study was conducted according to principles enshrined in the Declaration of Helsinki and in accordance with the Medical Research Involving Human Subjects Act (WMO).

## Availability of data and materials

The datasets generated during and/or analyzed during the current study are available in the appendices. Additional information can be obtained from the corresponding author on reasonable request.

## Funding

The study is partly funded by B. Braun Aesculap, they have no influence on any aspect of conception/writing of the protocol, selection of subjects, data acquisition or data analysis, or any step in the writing or publication process of articles concerning this study.

## Device(s)/drug(s)

Not applicable.

## Author contributions

1 guarantor of integrity of the entire study: VS, AS, IC, HvS, TB.

2. study concepts and design: VS, AS, IC, HvS, TB, 3. literature research: VS, AS, TB, 4. clinical studies: VS.

5 experimental studies/data analysis: VS, 6. TBstatistical analyses: VS, TB.

7 manuscript preparation: VS manuscript editing: AS, IC, HvS, TB.

## Declaration of competing interest

The authors declare that they have no known competing financial interests or personal relationships that could have appeared to influence the work reported in this paper.

## References

[bib1] AHO A., Vartiainen O., Salo O. (1955). Segmentary antero-posterior mobility of the cervical spine. Ann. Med. Intern. Fenn..

[bib2] Anderst W.J., Donaldson W.F., Lee J.Y., Kang J.D. (2014). Continuous cervical spine kinematics during in vivo dynamic flexion-extension. Spine J..

[bib3] Bhalla S.K., Simmons E.H. (1969). Normal ranges of intervertebral-joint motion of the cervical spine. Can. J. Surg..

[bib4] Bogduk N., Mercer S. (2000). Biomechanics of the cervical spine. I: normal kinematics. Clin. Biomech..

[bib5] Boselie T.F.M., Willems P.C., van Mameren H., de Bie R.A., Benzel E.C., van Santbrink H. (2013). Arthroplasty versus fusion in single-level cervical degenerative disc disease: a Cochrane review. Spine.

[bib6] Boselie T.F.M., van Mameren H., de Bie R.A., van Santbrink H. (2015). Cervical spine kinematics after anterior cervical discectomy with or without implantation of a mobile cervical disc prosthesis; an RCT. BMC Muscoskel. Disord..

[bib7] Boselie T.F.M., van Santbrink H., de Bie R.A., van Mameren H. (2017). Pilot study of sequence of segmental contributions in the lower cervical spine during active extension and flexion: healthy controls versus cervical degenerative disc disease patients. Spine.

[bib8] Boselie T.F.M., van Santbrink H., de Bie R.A., van Mameren H. (2017). Pilot study of sequence of segmental contributions in the lower cervical spine during active extension and flexion. Spine.

[bib9] Byvaltsev V.A., Stepanov I.A., Riew D.K. (2020). Mid-term to long-term outcomes after total cervical disk arthroplasty compared with anterior diskectomy and fusion: a systematic review and meta-analysis of randomized controlled trials. Clin spine Surg.

[bib10] Cooperation IBM. IBM SPSS Statistics for Windows. Version;.

[bib11] Dvorak J., Panjabi M.M., Novotny J.E., Antinnes J.A. (1991). In vivo flexion/extension of the normal cervical spine. J Orthop Res Off Publ Orthop Res Soc.

[bib12] Eck J.C., Humphreys S.C., Lim T.-H., Jeong S.T., Kim J.G., Hodges S.D. (2002). Biomechanical study on the effect of cervical spine fusion on adjacent-level intradiscal pressure and segmental motion. Spine.

[bib13] Findlay C., Ayis S., Demetriades A.K. (2018). Total disc replacement versus anterior cervical discectomy and fusion: a systematic review with meta-analysis of data from a total of 3160 patients across 14 randomized controlled trials with both short- and medium- to long-term outcomes. Bone Joint Lett. J.

[bib14] Hauerberg J., Kosteljanetz M., Bøge-Rasmussen T., Dons K., Gideon P., Springborg J.B. (2008). Anterior cervical discectomy with or without fusion with ray titanium cage: a prospective randomized clinical study. Spine.

[bib15] Helgeson M.D., Bevevino A.J., Hilibrand A.S. (2013). Update on the evidence for adjacent segment degeneration and disease. Spine J..

[bib16] Kong L., Cao J., Wang L., Shen Y. (2016). Prevalence of adjacent segment disease following cervical spine surgery. Méd..

[bib17] Lind B., Sihlbom H., Nordwall A., Malchau H. (1989). Normal range of motion of the cervical spine. Arch. Phys. Med. Rehabil..

[bib18] Martins A.N. (1976). Anterior cervical discectomy with and without interbody bone graft. J. Neurosurg..

[bib19] NCT04545983 (2020). NCT04545983.

[bib20] R K., C D. (2016). Five-year clinical results of cervical total disc replacement compared with anterior discectomy and fusion for treatment of 2-level symptomatic degenerative disc disease: a prospective, randomized, controlled, multicenter investigational device exemption. J. Neurosurg. Spine.

[bib21] Reinartz R., Platel B., Boselie T., van Mameren H., van Santbrink H., Romeny B. ter H. (2009). Cervical vertebrae tracking in video-fluoroscopy using the normalized gradient field. Med image Comput Comput Interv MICCAI. Int Conf Med Image Comput Comput Interv.

[bib22] Schuermans V.N.E., Smeets A.Y.J.M., Boselie T.F.M., Candel M.J.J.M., Curfs I., Evers S.M.A.A. (2022). Research protocol: cervical Arthroplasty Cost Effectiveness Study (CACES): economic evaluation of anterior cervical discectomy with arthroplasty (ACDA) versus anterior cervical discectomy with fusion (ACDF) in the surgical treatment of cervical degenerat. Trials.

[bib23] Schuermans V.N.E., Smeets A.Y.J.M., Curfs I., van Santbrink H., Boselie T.F.M. (2023).

[bib24] Schuermans V, Smeets AYJM, Wijsen NPMH, Curfs I, T.F.M. B, van Santbrink H. Clinical Adjacent Segment Pathology after Anterior Cervical Decompression Surgery for Cervical Degenerative Disc Disease: a Single Center Retrospective Cohort Study with Long-Term Follow-Up.. 10.1016/j.bas.2022.100869 PMC9560678 36248168

[bib25] Schuermans VNE, Breen A, Branney J, Smeets AYJM, van Santbrink H, Boselie TFM. Cross-validation of Two Independent Methods to Analyze the Sequence of Segmental Contributions in the Cervical Spine in Extension Cineradiographic Recordings.. 10.1186/s12891-025-09413-1 PMC12822052 41398254

[bib26] Seo M., Choi D. (2008). Adjacent segment disease after fusion for cervical spondylosis; myth or reality?. Br. J. Neurosurg..

[bib27] Tsang S.M.H., Gpy Szeto, Lee R.Y.W. (2014). Altered spinal kinematics and muscle recruitment pattern of the cervical and thoracic spine in people with chronic neck pain during functional task. J Electromyogr Kinesiol Off J Int Soc Electrophysiol Kinesiol.

[bib28] van Mameren H. (1988).

[bib29] Van Mameren H., Drukker J., Sanches H., Beursgens J. (1990). Cervical spine motion in the sagittal plane (I) range of motion of actually performed movements, an X-ray cinematographic study. Eur. J. Morphol..

[bib30] Vleggeert-Lankamp C.L.A., Janssen T.M.H., van Zwet E., Goedmakers C.M.W., Bosscher L., Peul W. (2019). The NECK trial: effectiveness of anterior cervical discectomy with or without interbody fusion and arthroplasty in the treatment of cervical disc herniation; a double-blinded randomized controlled trial. Spine J..

[bib31] Wan Z., Wang W., Li C., Li J., Lin J., Tian F. (2021). Validation and application of a novel in vivo cervical spine kinematics analysis technique. Sci. Rep..

[bib32] Wu J.-C., Chang H.-K., Huang W.-C., Tu T.-H., Fay L.-Y., Kuo C.-H. (2019). Radiological and clinical outcomes of cervical disc arthroplasty for the elderly: a comparison with young patients. BMC Muscoskel. Disord..

[bib33] Xie J., Hurlbert R.J. (2007). Discectomy versus discectomy with fusion versus discectomy with fusion and instrumentation: a prospective randomized study. Neurosurgery.

[bib34] Yeni Y.N., Azad S., Oravec D., Schildcrout A., Basheer A., Bey M.J. (2022). Intervertebral kinematics during neck motion 6.5 years after fusion and artificial disc replacement. Clin. Biomech..

[bib35] Zavras A.G., Dandu N., Nolte M.T., Butler A.J., Federico V.P., Sayari A.J. (2022). Segmental range of motion after cervical total disc arthroplasty at long-term follow-up: a systematic review and meta-analysis. J. Neurosurg. Spine.

[bib36] Zhang Y., Liang C., Tao Y., Zhou X., Li H., Li F. (2015). Cervical total disc replacement is superior to anterior cervical decompression and fusion: a meta-analysis of prospective randomized controlled trials. PLoS One.

