# Supplementary material for: A randomized controlled trial with extended long-term follow-up: Quality of cervical spine motion after anterior cervical discectomy (ACD) or anterior cervical discectomy with arthroplasty (ACDA)
Source: Brain Spine. 2023 Dec 14;4:102726. doi: 10.1016/j.bas.2023.102726 (PMC10951699; doi:10.1016/j.bas.2023.102726)
Supplement: Multimedia component 1 [file mmc1.pdf]

P1 | **Pre-operative** | **1- year follow-up** | **Long term follow-up**

ACDA C5-C6

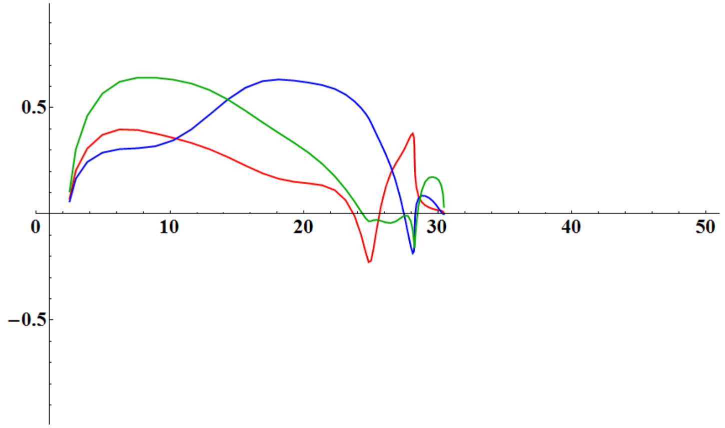

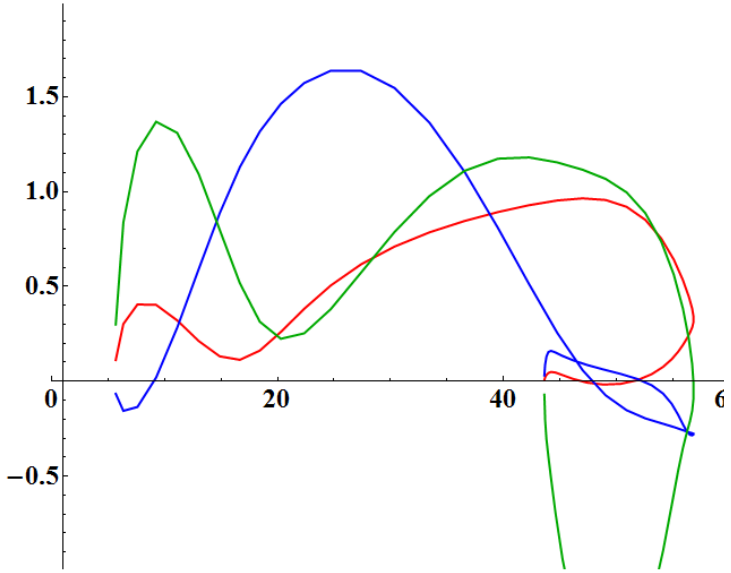

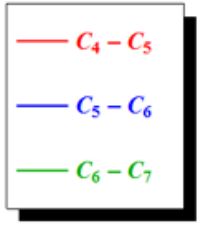

Y-axis: relative rotation of the individual segment
X-axis: segment C4-C7

P2 | Pre-operative | 1- year follow-up | Long term follow-up

ACDA
C5-C6

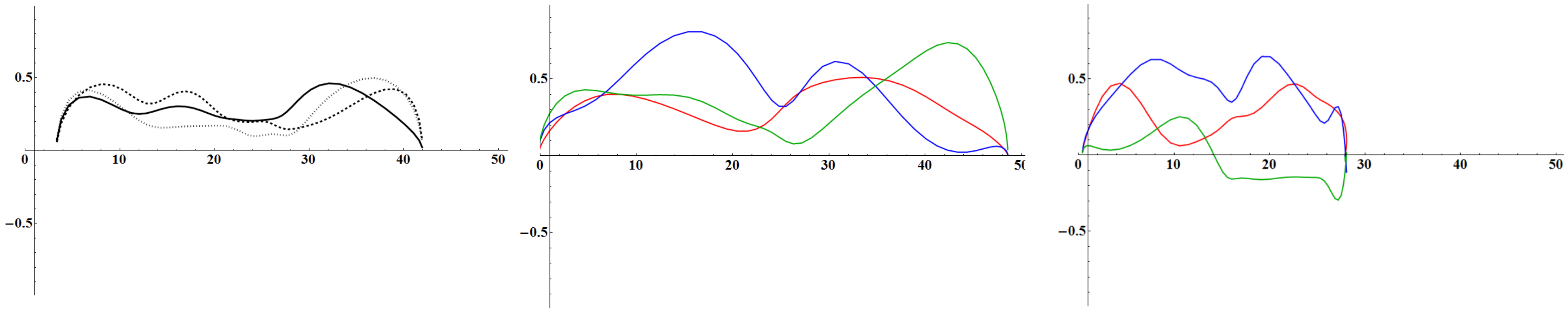

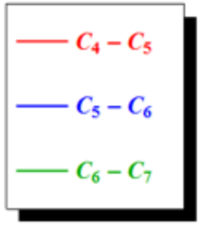

Y-axis: relative rotation of the individual segment
X-axis: segment C4-C7

P3

ACDA C6-C7

Pre-operative

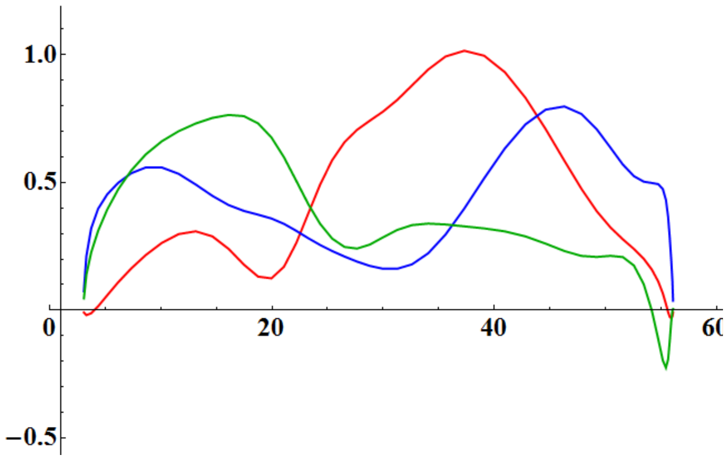

1- year follow-up

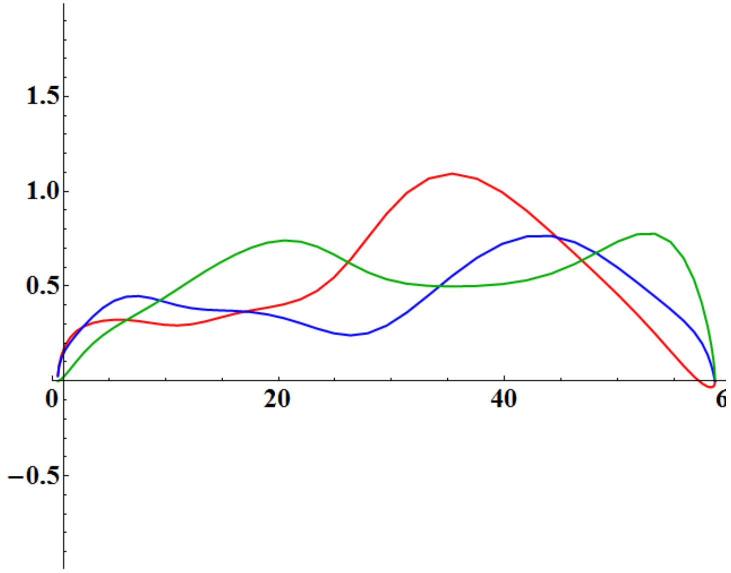

Long term follow-up

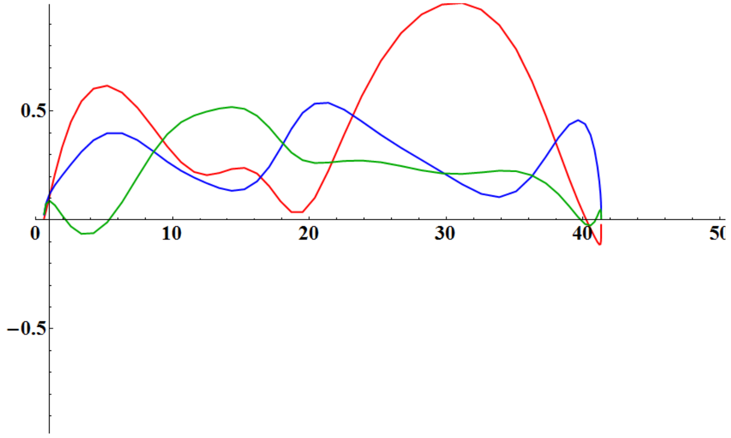

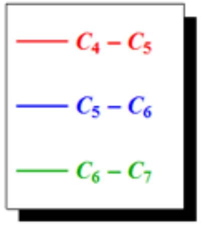

Y-axis: relative rotation of the individual segment
X-axis: segment C4-C7

P4 **Pre-operative** **1- year follow-up** **Long term follow-up**

ACDA C5-C6

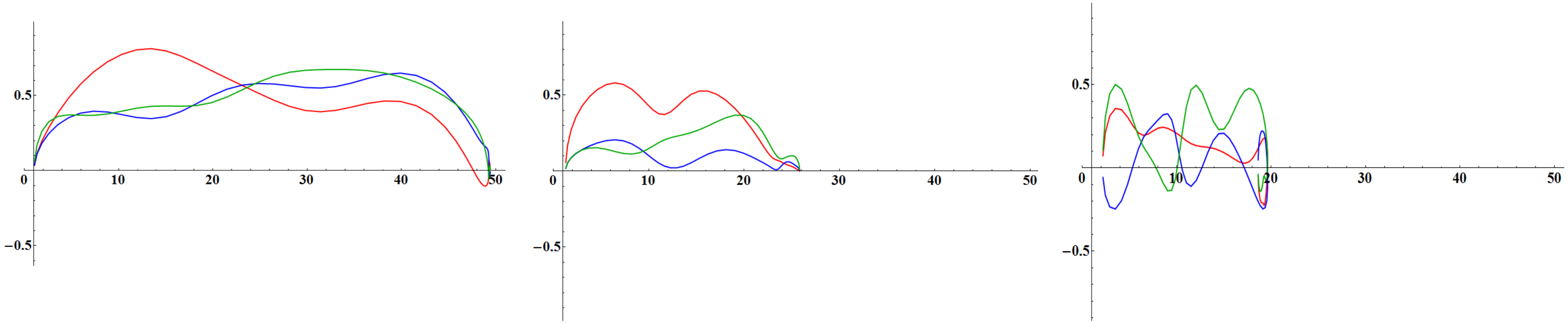

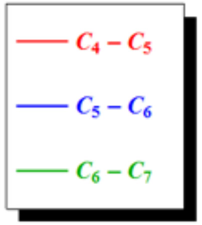

Y-axis: relative rotation of the individual segment
X-axis: segment C4-C7

P5 **Pre-operative** **1- year follow-up** **Long term follow-up**

ACDA C5-C6

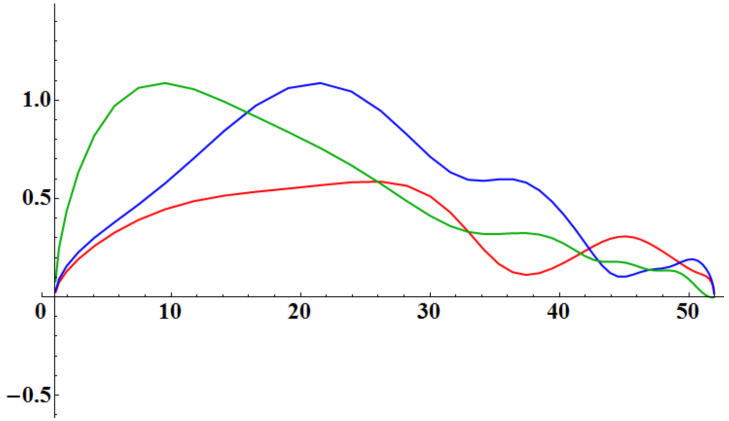

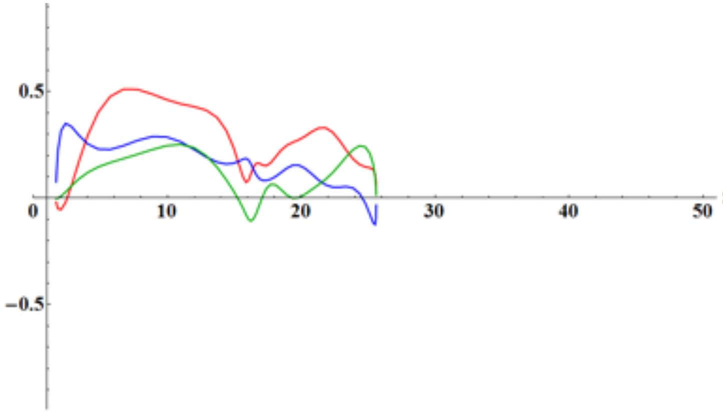

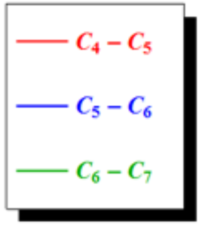

Y-axis: relative rotation of the individual segment
X-axis: segment C4-C7

P6 | Pre-operative | 1- year follow-up | Long term follow-up

ACD
C5-C6

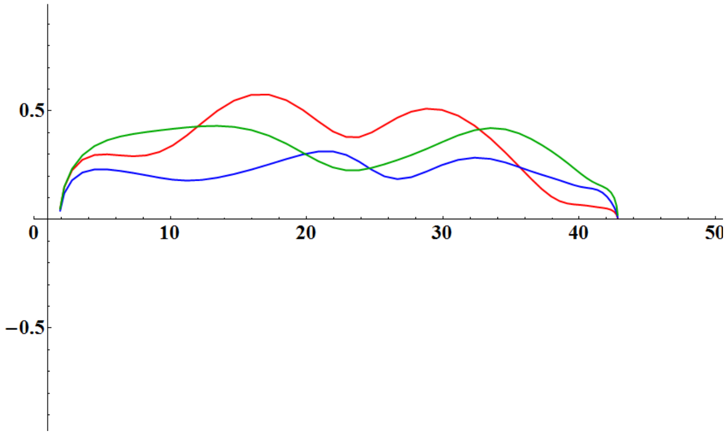

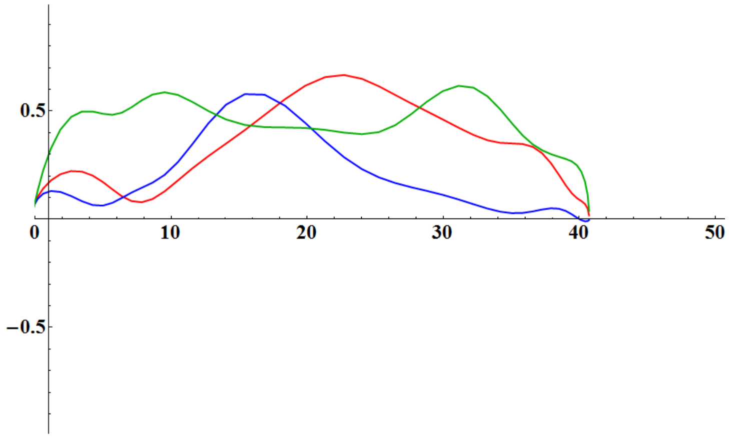

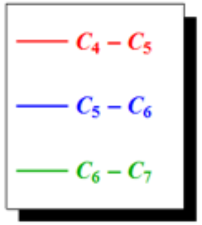

Y-axis: relative rotation of the individual segment
X-axis: segment C4-C7

P7 **Pre-operative** **1- year follow-up** **Long term follow-up**

ACD
C6-C7

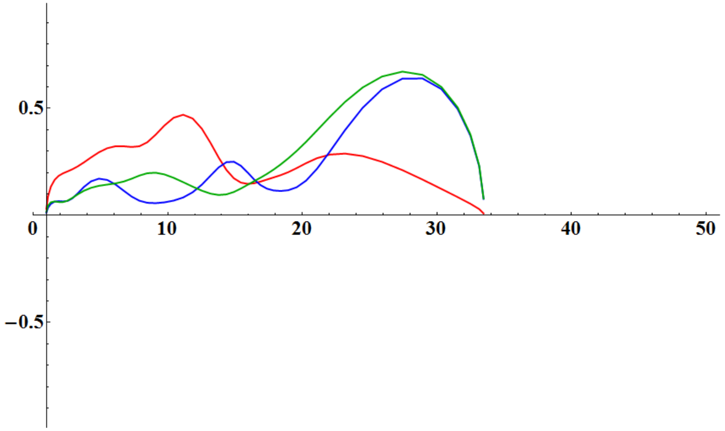

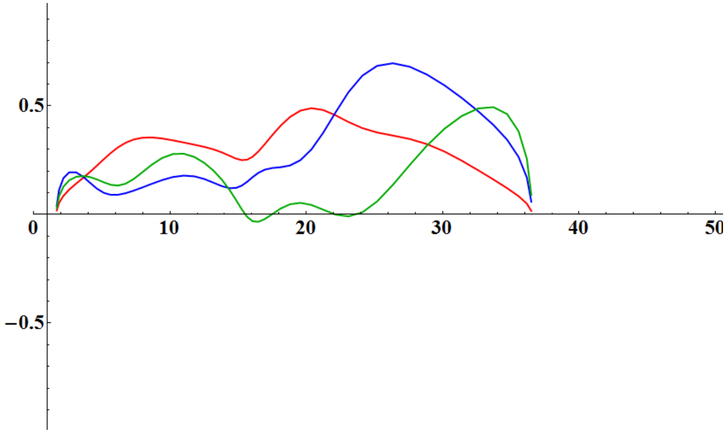

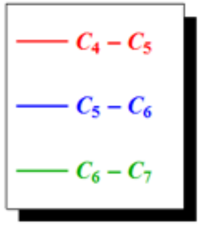

Y-axis: relative rotation of the individual segment
X-axis: segment C4-C7

P8 | Pre-operative | 1- year follow-up | Long term follow-up

ACD C5-C6

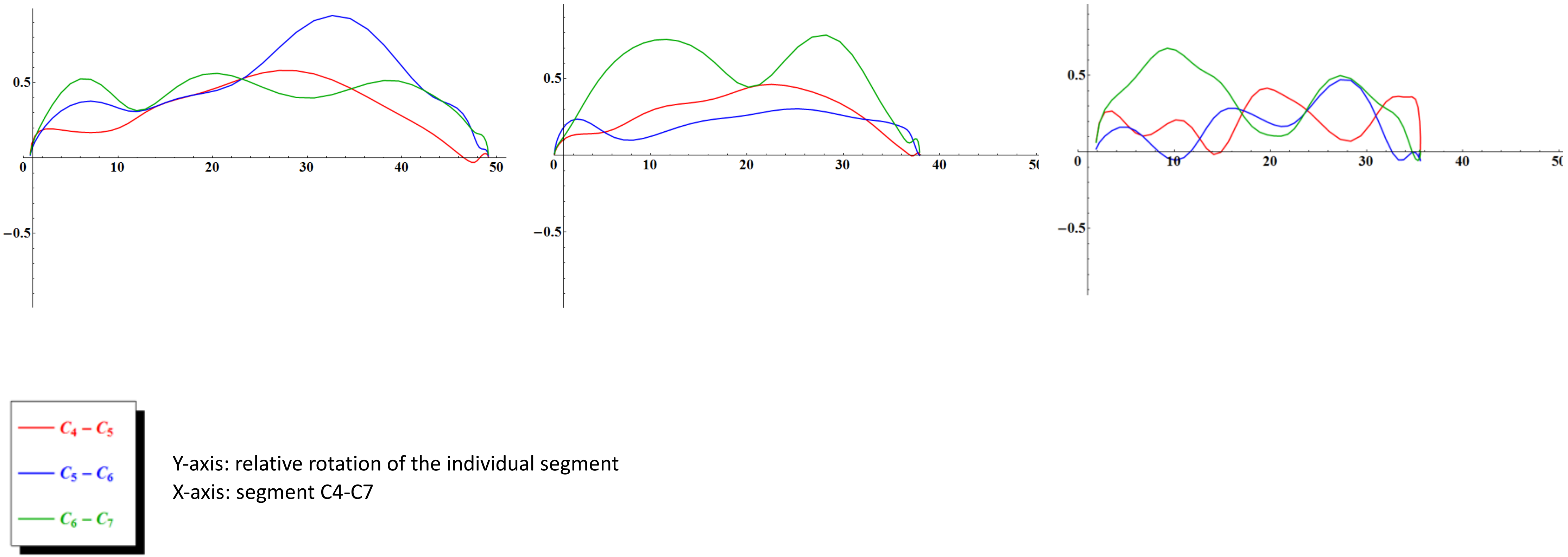

Y-axis: relative rotation of the individual segment
X-axis: segment C4-C7

P9 **Pre-operative** **1- year follow-up** **Long term follow-up**

ACD C6-C7

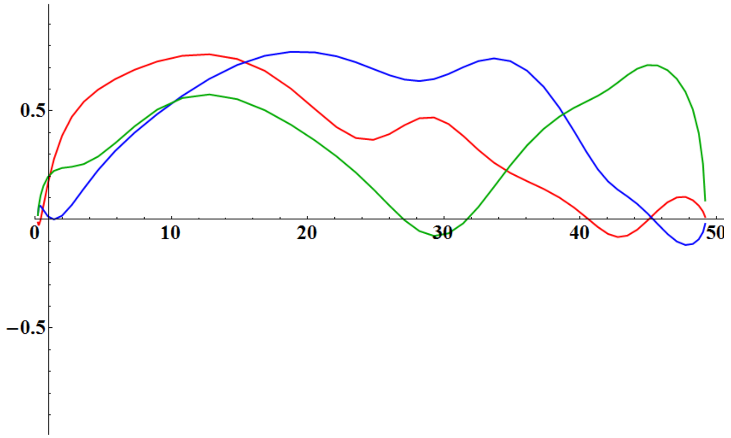

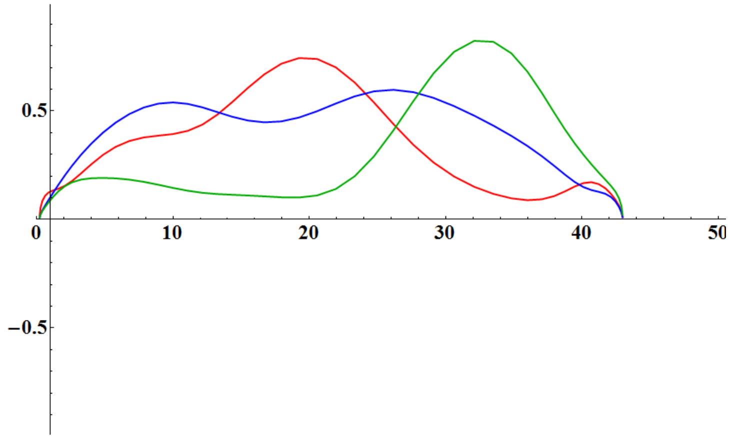

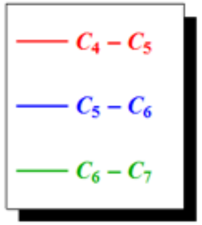

Y-axis: relative rotation of the individual segment
X-axis: segment C4-C7

P10 **Pre-operative** **1- year follow-up** **Long term follow-up**

ACD C6-C7

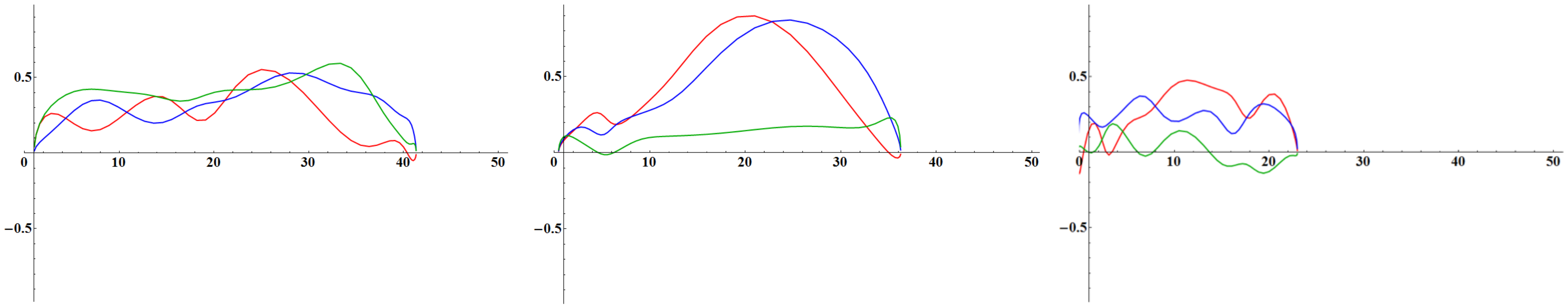

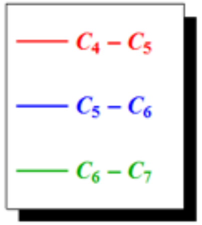

Y-axis: relative rotation of the individual segment
X-axis: segment C4-C7

P11 **Pre-operative** **1- year follow-up** **Long term follow-up**

ACD C6-C7

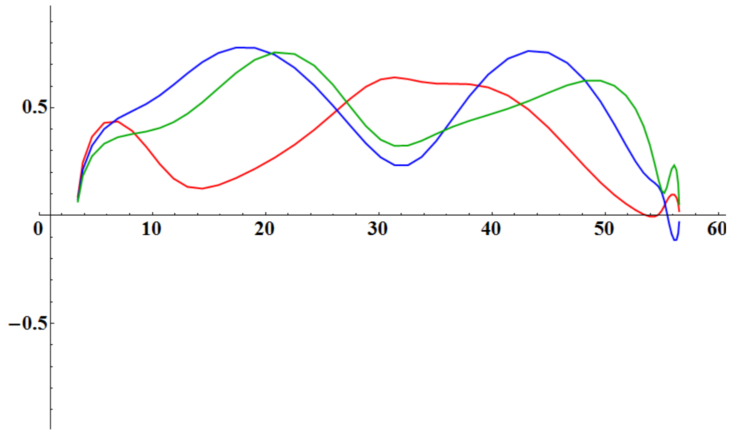

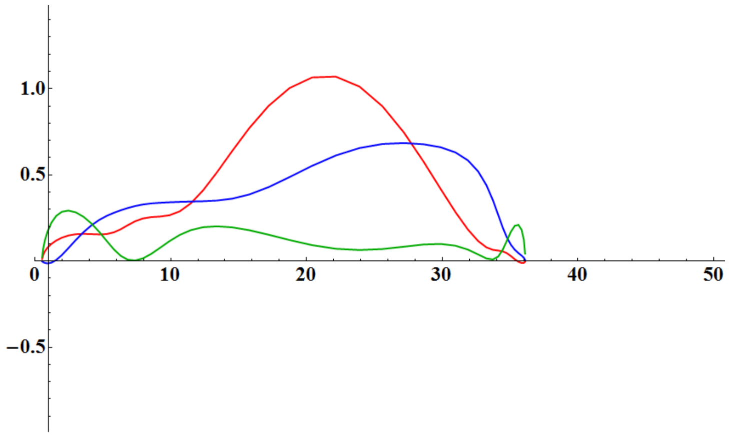

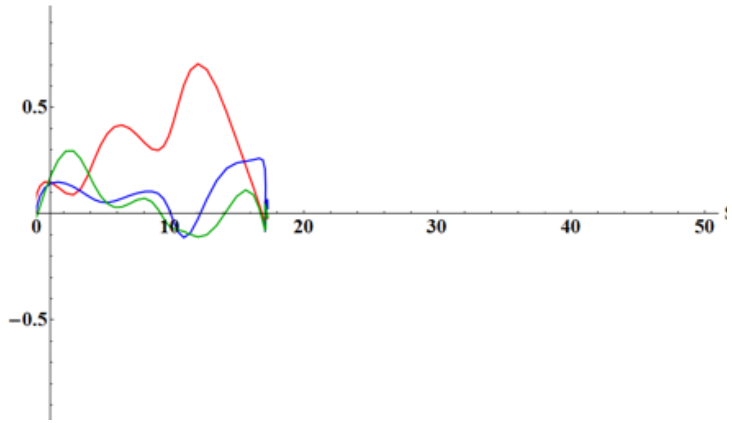

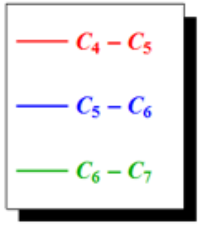

Y-axis: relative rotation of the individual segment
X-axis: segment C4-C7

P12 | **Pre-operative** | **1- year follow-up** | **Long term follow-up**

ACDA C6-C7

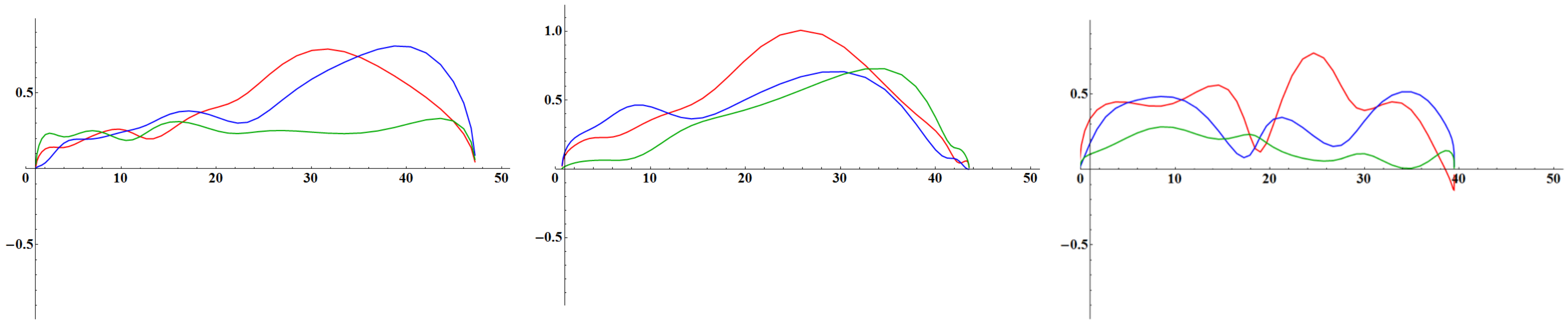

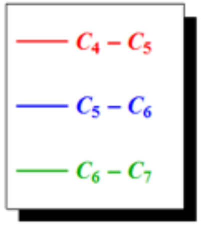

Y-axis: relative rotation of the individual segment
X-axis: segment C4-C7

P13 **Pre-operative** **1- year follow-up** **Long term follow-up**

ACDA
C5-C6

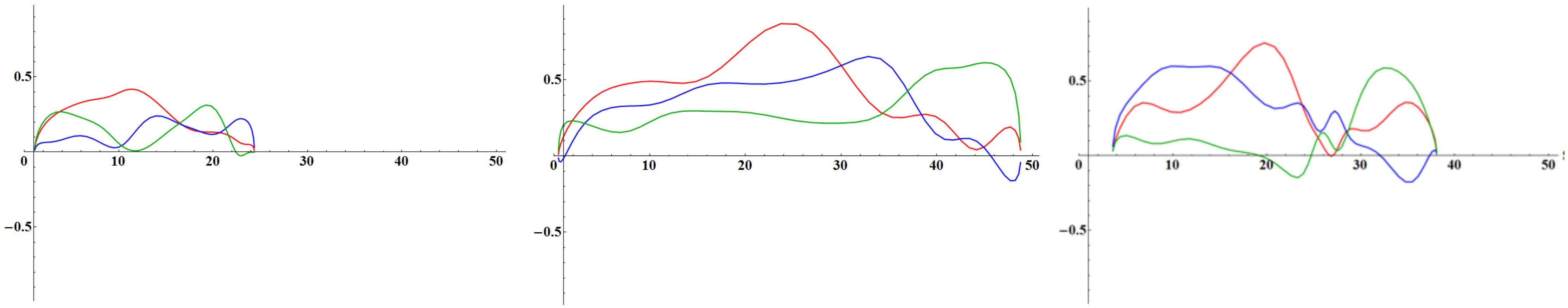

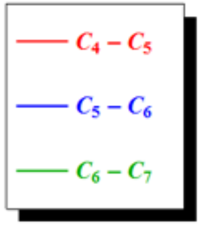

Y-axis: relative rotation of the individual segment
X-axis: segment C4-C7

P14 | **Pre-operative** | **1- year follow-up** | **Long term follow-up**

ACD C6-C7

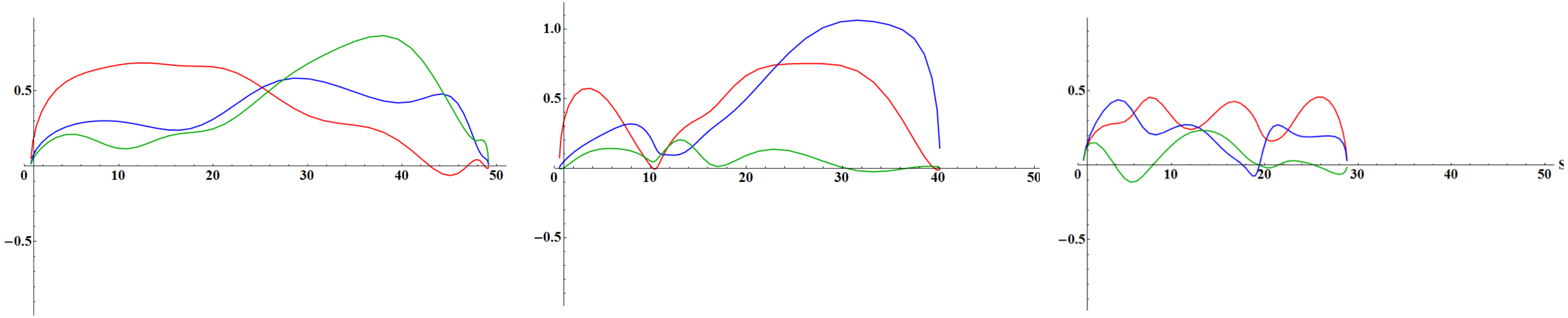

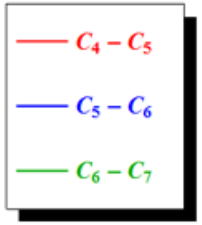

Y-axis: relative rotation of the individual segment
X-axis: segment C4-C7

P15 **Pre-operative** **1- year follow-up** **Long term follow-up**

ACDA C5-C6

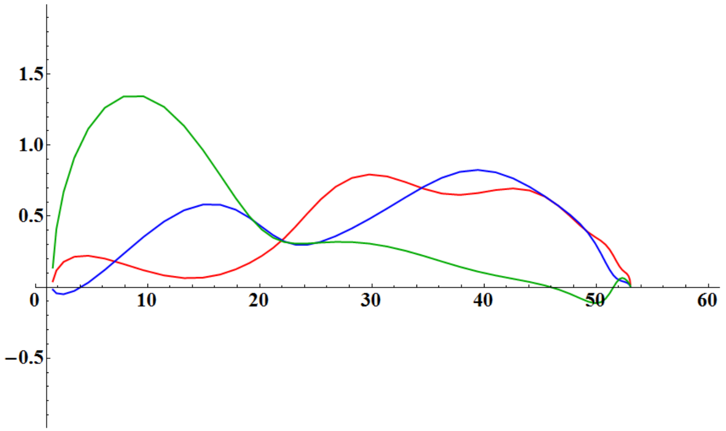

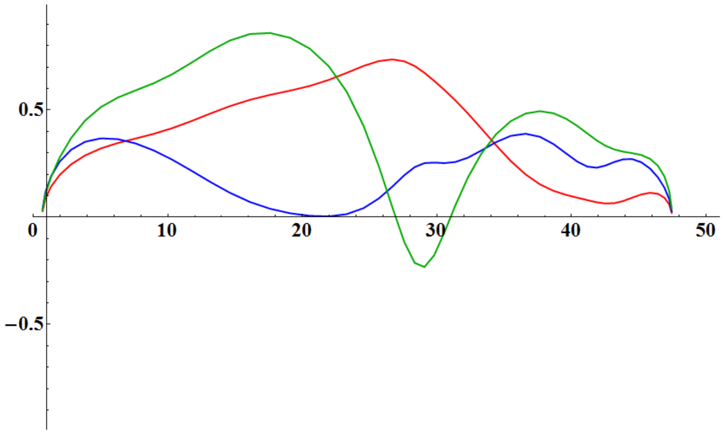

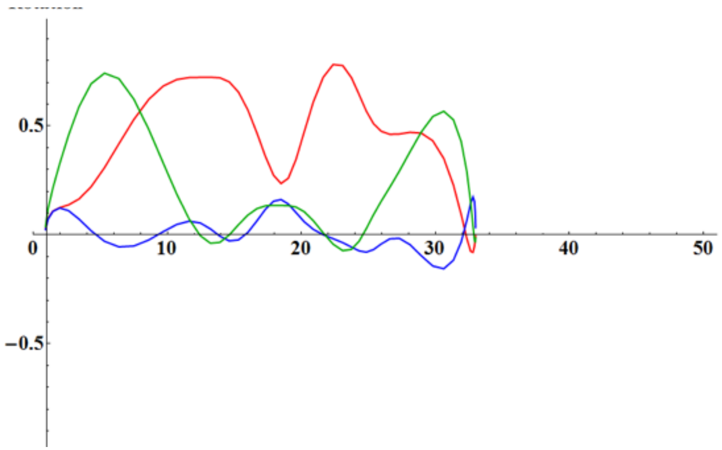

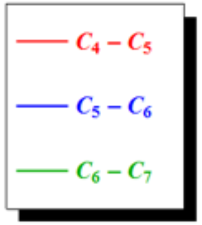

Y-axis: relative rotation of the individual segment
X-axis: segment C4-C7

P16 **Pre-operative** **1- year follow-up** **Long term follow-up**

ACD
C5-C6

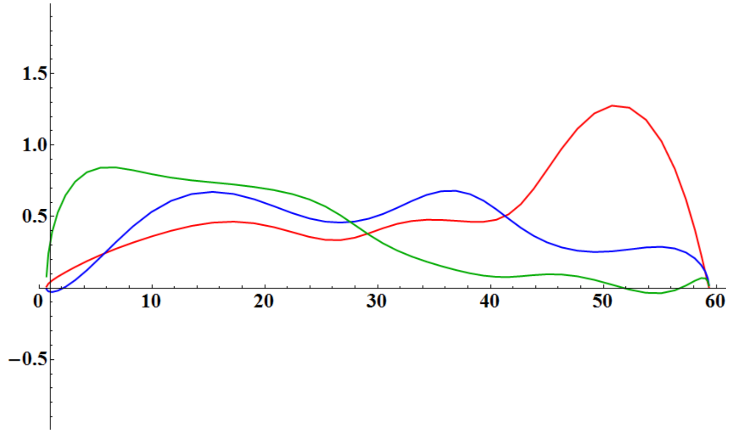

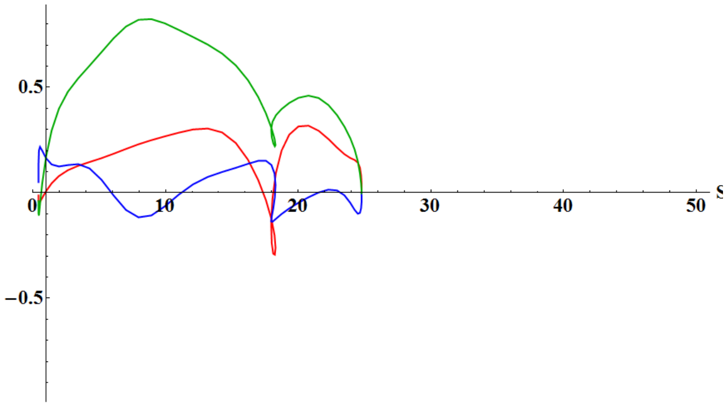

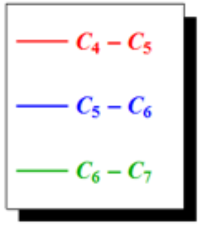

Y-axis: relative rotation of the individual segment
X-axis: segment C4-C7

P17 **Pre-operative** **1- year follow-up** **Long term follow-up**

ACDA C5-C6

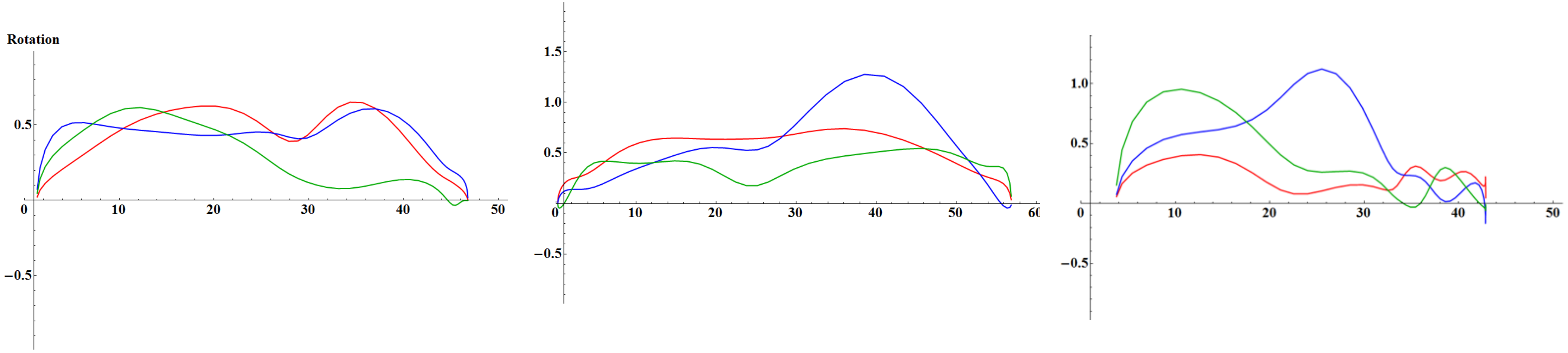

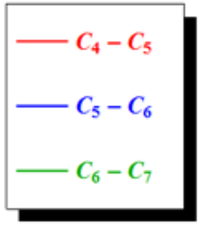

Y-axis: relative rotation of the individual segment
X-axis: segment C4-C7

P18

Pre-operative

1- year follow-up

Long term follow-up

ACD
C6-C7

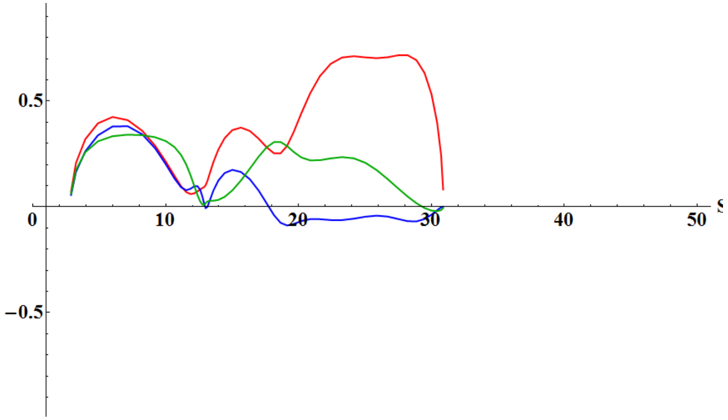

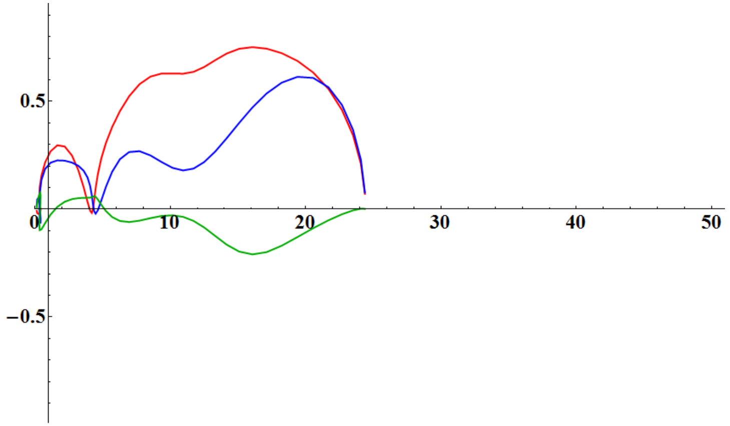

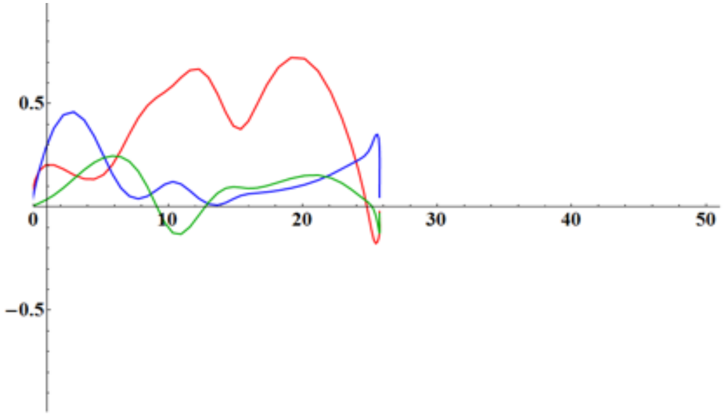

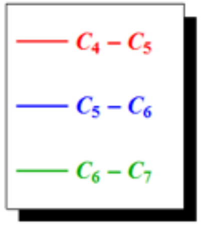

Y-axis: relative rotation of the individual segment
X-axis: segment C4-C7

P19 **Pre-operative** **1- year follow-up** **Long term follow-up**

ACD C5-C6

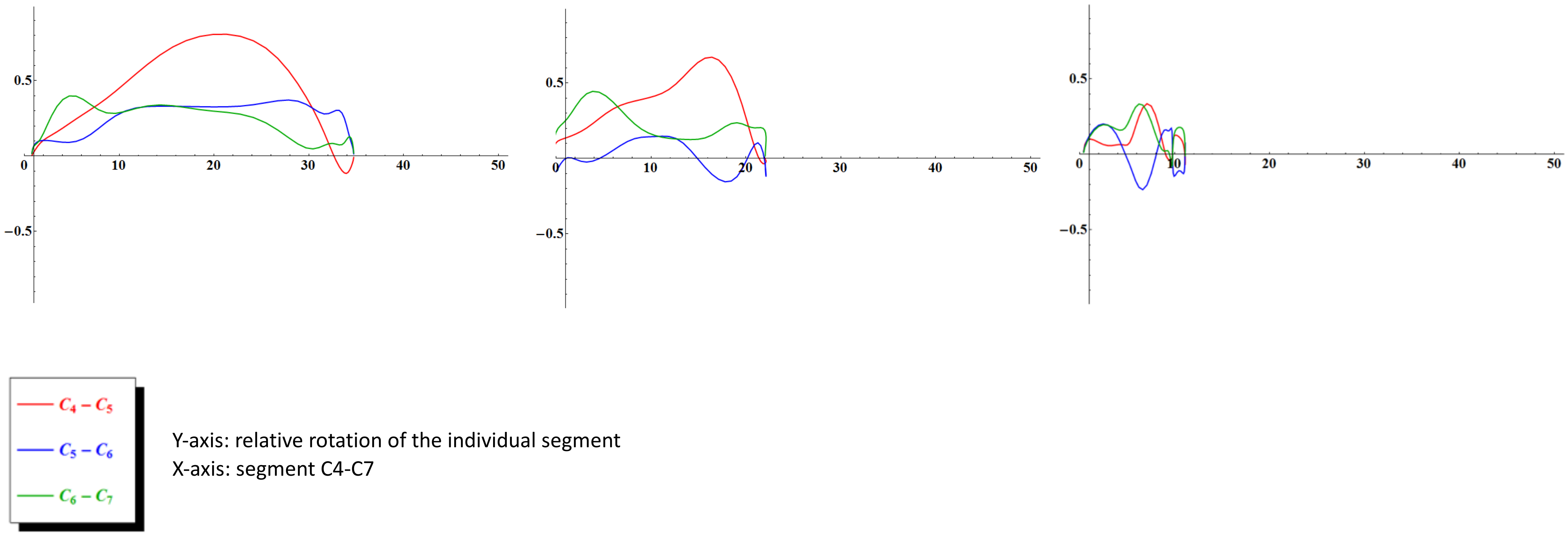

Y-axis: relative rotation of the individual segment
X-axis: segment C4-C7

P20 **Pre-operative** **1- year follow-up** **Long term follow-up**

ACD
C5-C6

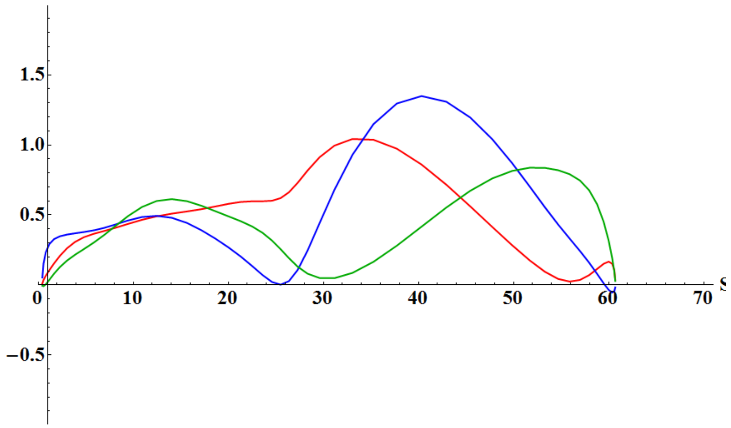

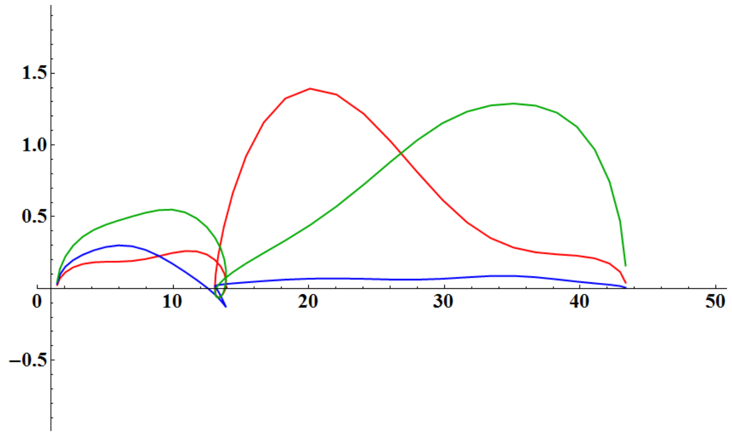

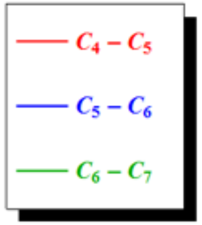

Y-axis: relative rotation of the individual segment
X-axis: segment C4-C7

P21 | Pre-operative | 1- year follow-up | Long term follow-up

ACDA C5-C6

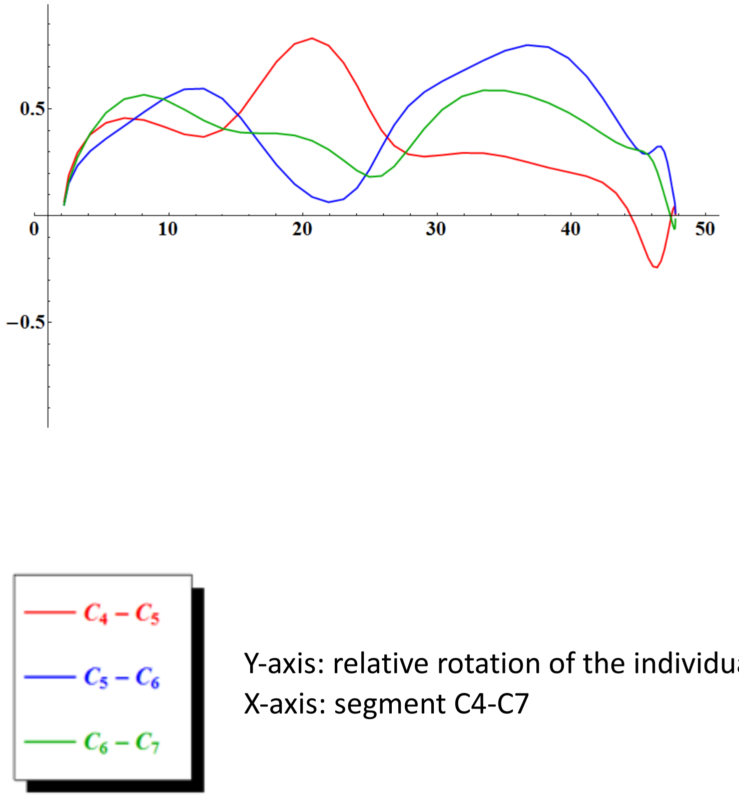

Y-axis: relative rotation of the individual segment
X-axis: segment C4-C7

P22

Pre-operative

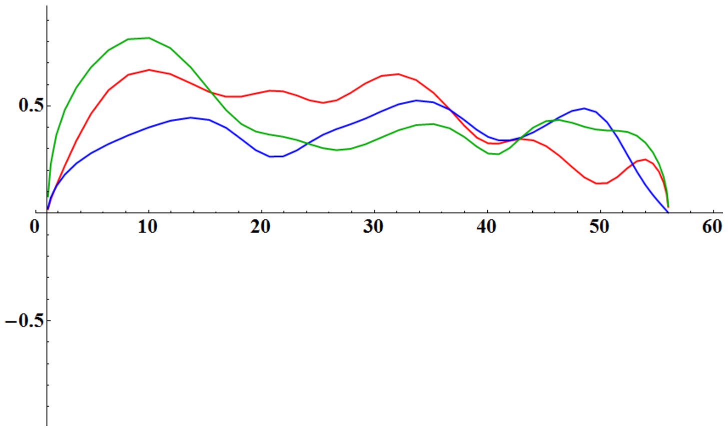

1- year follow-up

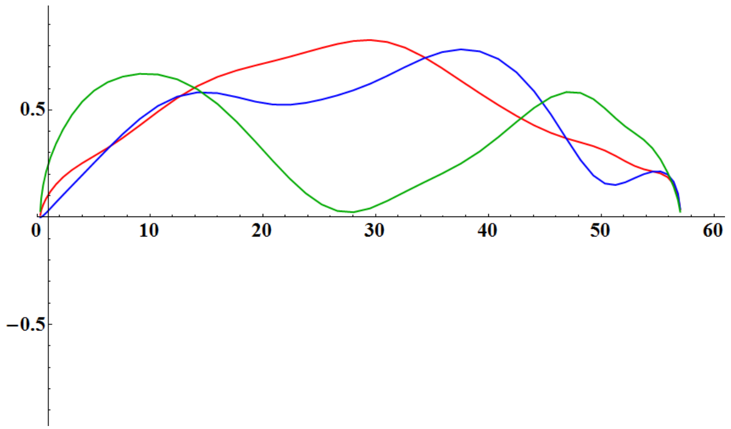

Long term follow-up

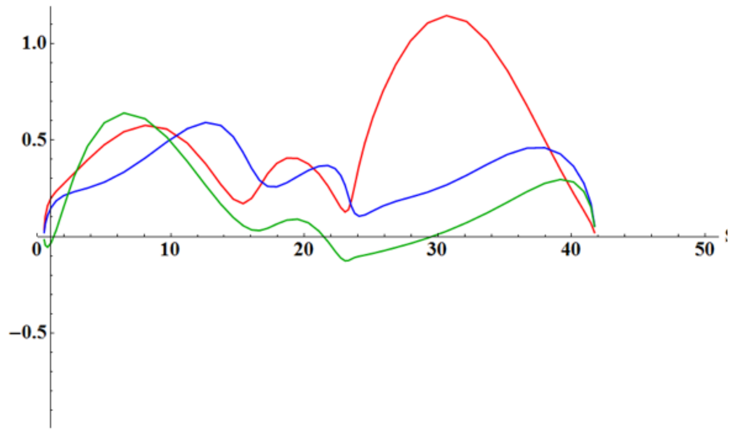

ACDA C5-C6

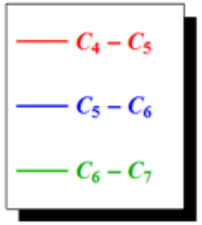

Y-axis: relative rotation of the individual segment
X-axis: segment C4-C7

P23

ACDA C5-C6

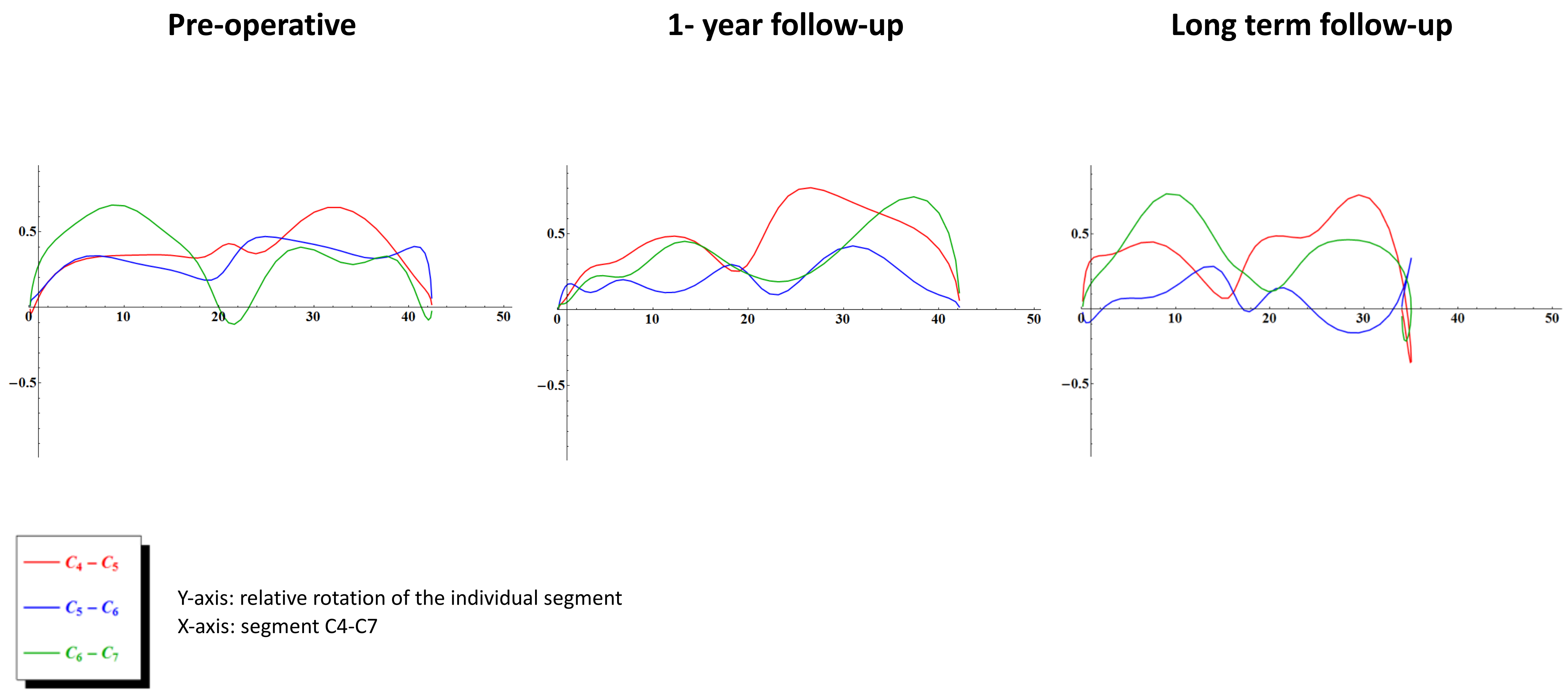

Y-axis: relative rotation of the individual segment
X-axis: segment C4-C7

P24	Pre-operative	1- year follow-up	Long term follow-up

ACDA C5-C6

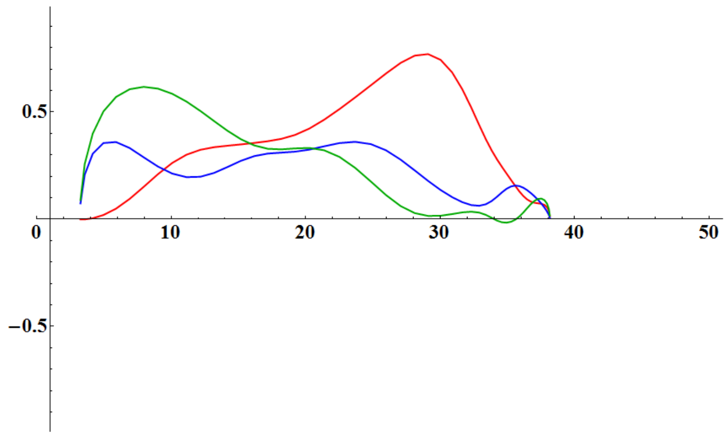

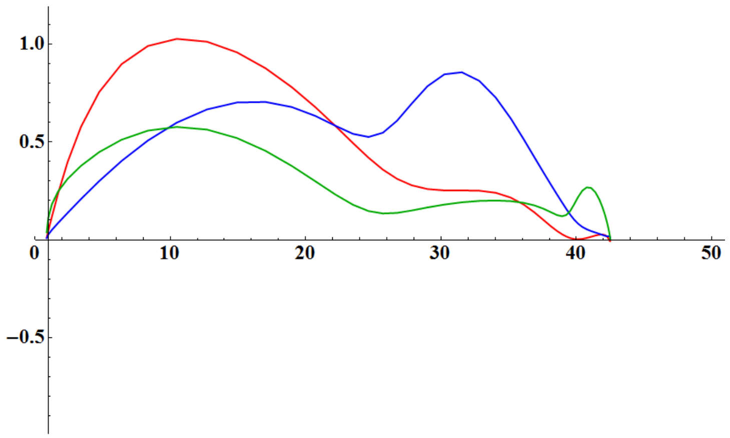

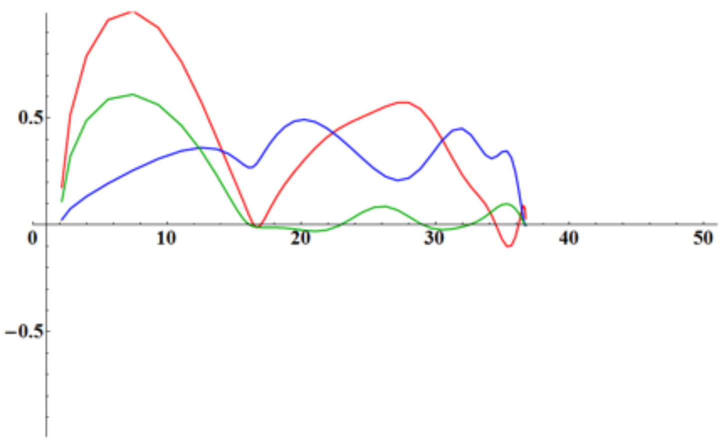

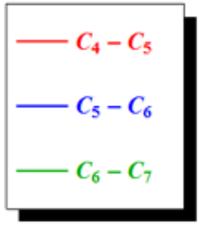

Y-axis: relative rotation of the individual segment
X-axis: segment C4-C7

P25 **Pre-operative** **1- year follow-up** **Long term follow-up**

ACD
C5-C6

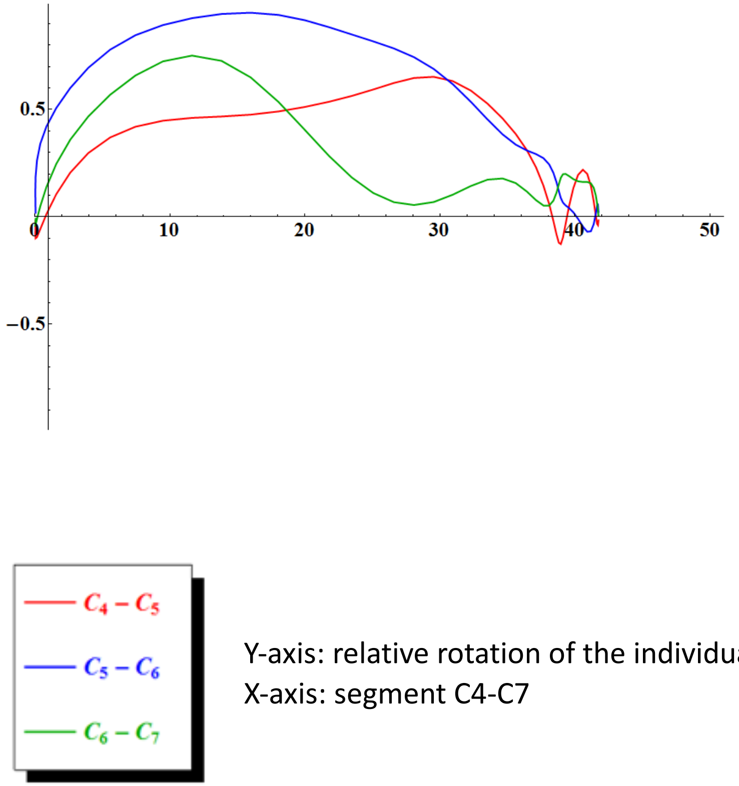

Y-axis: relative rotation of the individual segment
X-axis: segment C4-C7

P26 | Pre-operative | 1- year follow-up | Long term follow-up

ACDA
C6-C7

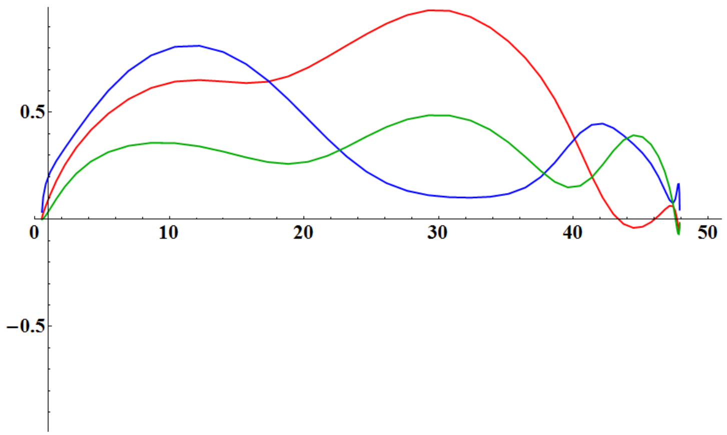

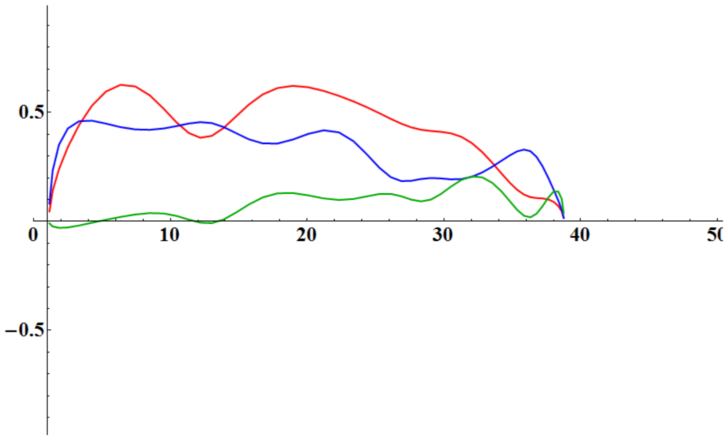

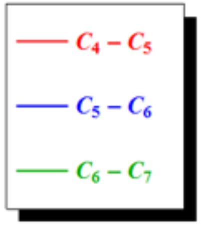

Y-axis: relative rotation of the individual segment
X-axis: segment C4-C7

P27 **Pre-operative** **1- year follow-up** **Long term follow-up**

ACDA
C6-C7

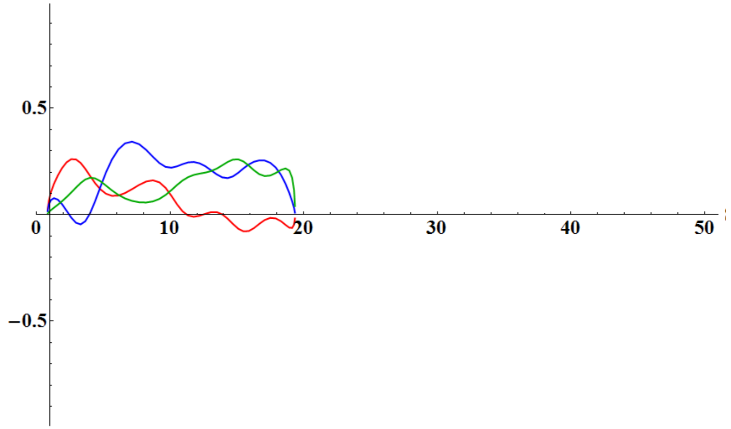

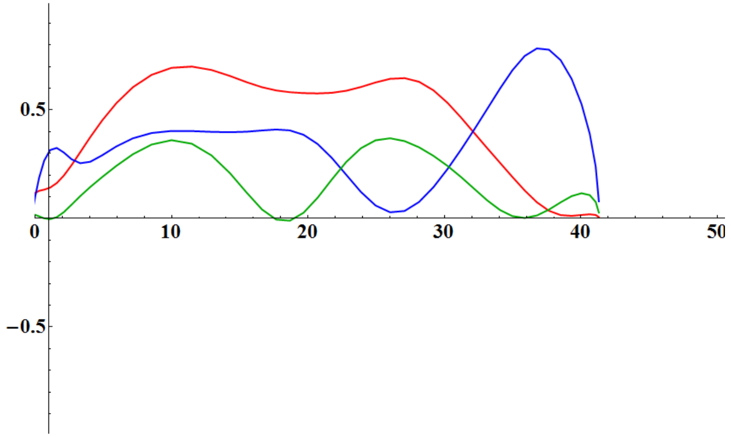

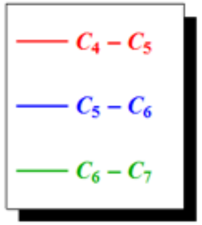

Y-axis: relative rotation of the individual segment
X-axis: segment C4-C7
