# Supplementary material for: A randomized controlled trial with extended long-term follow-up: Quality of cervical spine motion after anterior cervical discectomy (ACD) or anterior cervical discectomy with arthroplasty (ACDA)
Source: Brain Spine. 2023 Dec 14;4:102726. doi: 10.1016/j.bas.2023.102726 (PMC10951699; doi:10.1016/j.bas.2023.102726)
Supplement: Multimedia component 2 [file mmc2.docx]

Appendix 2: Ranges of motion per individual segment expressed in degrees, for both one-year and extended follow-up. tROM = total range of motion from C4-C7, sROM = segmental range of motion, SD = standard deviation.

|  | | **tROM (˚)**  **C4-C7** | **sROM (˚)**  **C4-C5** | **sROM (˚)**  **C5-C6** | **sROM (˚)**  **C6-C7** | **sROM index level** | **sROM upper AS** | **sROM lower AS** |
| --- | --- | --- | --- | --- | --- | --- | --- | --- |
| **1-year** | **ACD ± SD (N=10)** | **35.1 ± 6.6 *** | 14.4 ± 2.6 | 10.2 ± 6.9 | 9.3 ± 8.5 | **4.5 ± 3.3 *** | 14.0 ± 2.7 | 17.8 ± 5.3 |
|  | **ACDA ± SD (N=13)** | **45.1 ± 9.7*** | 17.4 ± 3.0 | 13.3 ± 5.5 | 13.9 ± 3.4 | **13.8 ± 5.1 *** | 16.8 ± 2.5 | 13.8 ± 4.1 |
| **11-years** | **ACD ± SD (N=7)** | **22.2 ± 7.0 *** | **9.9 ± 4.3 *** | **5.9 ± 4.0 *** | 6.4 ± 7.3 | **2.06 ± 2.21 *** | **7.37 ± 3.03 *** | 13.00 ± 6.61 |
|  | **ACDA ± SD (N=11)** | **31.8 ± 7.7 *** | **14.0 ± 4.6 *** | **10.5 ± 5.8 *** | 7.3 ± 4.4 | **9.38 ± 5.69 *** | **13.32 ± 4.41 *** | 7.15 ± 4.84 |
